# Assessing maternal and newborn health readiness: Insights from a service availability assessment in five provinces in Laos

**DOI:** 10.1371/journal.pone.0331659

**Published:** 2025-09-11

**Authors:** Elizabeth Bunde, Elena Herrera, Lathtana Chanthala, Jessica Posner, Douangphone Sohkivong

**Affiliations:** 1 John Snow, Inc., Vientiane, Lao People’s Democratic Republic; 2 John Snow, Inc., Washington, District of Columbia, United States of America; 3 Department of Healthcare and Rehabilitation, Ministry of Health, Vientiane, Lao People’s Democratic Republic; Donor Organization in Nepal, NEPAL

## Abstract

**Background:**

Global maternal mortality rates have declined significantly over the past two decades, including an 80% reduction in Laos since 2000. Effective management of obstetric complications – a major contributor to maternal deaths – requires well-staffed facilities equipped with essential supplies, medicines, and infrastructure. Despite progress, Laos still faces gaps in service availability and readiness limiting further reductions in preventable maternal mortality.

**Objective:**

This analysis aimed to assess the service availability and readiness of public health facilities in five provinces of Laos to deliver maternal and newborn healthcare, including basic emergency obstetric and newborn care services (BEmONC).

**Methods:**

A cross-sectional survey was conducted In October-November 2023 across 212 health centers and 20 district hospitals under the Laos Maternal Child Health and Nutrition project. Descriptive analysis was used to analyze the data. Service availability was measured based on the number of facilities and beds relative to the population. Service readiness was measured across three domains: guidelines and trained staff, essential equipment and supplies, and essential medicines. A composite readiness score was calculated as the mean across these domains. BEmONC availability was assessed using the presence of seven signal functions.

**Findings:**

The overall service availability score was 71.3% across all provinces. The antenatal care readiness score across both facility types was 69.0%, CI_95_:62.7–75.3%, with district hospitals scoring higher than health centers, at 77.1%, CI_95%_:75.4–78.8%, compared to 68.2%, CI_95%_: 62.1–4.3%. The mean readiness score for delivery and newborn care was 68.3%, CI_95%_: 59.5–77.1%, with district hospitals again performing better at 81.3%, CI_95%_:79.7–82.9% compared to 67.0%, CI_95%_:58.2–75.8% for health centers.

**Conclusion:**

Critical gaps in maternal and newborn health services remain, particularly in health centers. Investments in staffing, infrastructure, and availability of equipment and medicines is essential to address current gaps, improve service readiness, and contribute to improved quality of care and health outcomes.

## Introduction

Over the past two decades, there have been substantial improvements in global maternal mortality rates (MMR), decreasing by 33% globally and by 39% in East and Southeast Asia [1]. Despite these gains, progress has been insufficient to reach the target of less than 70 deaths per 100,000 live births set in the Sustainable Development Goal (SDG) [2]. Additionally, MMRs have largely stagnated in recent years with over 800 maternal deaths occurring daily [1]. Most maternal deaths result from complications arising during pregnancy, childbirth, or the postpartum period that, with timely medical intervention and proper healthcare infrastructure, could be largely prevented [3].

To reduce preventable maternal deaths, a renewed focus on effective strategies throughout pregnancy, childbirth, and the postpartum period is essential. The leading direct obstetric causes—hemorrhage, sepsis, obstructed labor, and pre-eclampsia or eclampsia—are largely preventable with timely medical intervention [4–6]. Previous studies have identified several barriers to accessing maternal healthcare. Among clients, geographic location and the cost and availability of transportation are significant obstacles to accessing maternal health services [7,8]. At facilities, inadequate healthcare infrastructure, limited medical supplies, and shortages of essential medicines and commodities can hinder the timely delivery of quality obstetric care [9,10].

Between 2000 and 2020, Laos has reduced MMR by approximately 80%, from 579 to 126 maternal deaths per 100,00 live births, with corresponding reductions in the infant mortality rate from 86.8 to 39.0 deaths per 1,000 live births those same years [1,11]. Contributing to these reductions have been significant investments in health financing, service delivery, and related health infrastructure. This has resulted in increased use of skilled birth attendants from 64.4 to 79.8%, facility-based births from 64.5 to 78.2%, four antenatal care (ANC) visits from 62.2 to 71.6%, and postnatal care for newborns from 47.1 to 64.0% between 2017 and 2023 [12,13]. Additionally, the launch of the National Health Insurance (NHI) scheme in 2016 helped strengthen and broaden access to free maternal child health services, along with other protection measures, to expand coverage [14].

Laos has a decentralized healthcare system largely driven by the public sector, though the private sector is gradually expanding [15]. Health centers serve as the frontline for primary care, offering preventive services and treatment, and typically act as the first point of contact for patients in the public system. More complex cases are referred to district or provincial hospitals, which provide more advanced care. Despite the essential role of health centers, many people bypass them in favor of district or provincial hospitals, which are better equipped and seen as providing higher-quality services. This has also led to a perception that public healthcare, especially at the primary care level, is subpar compared to private options [16]. Despite this perception, maternal and newborn service uptake is still predominantly in the public sector. Recent 2023 data indicate that the majority of births were delivered in public facilities at 77.7% compared to private facilities at 0.6% [17]. Similarly, 89.1% of post-natal care visits within one week of birth were completed at public facilities compared to 1.5% at private facilities [17].

Despite significant strides in healthcare and development, Laos continues to face major challenges in maternal health. Laos still has one of the highest MMRs in the Southeast Asia region, with uneven progress across geographic regions and ethnic and socioeconomic groups [1,18,19]. Ensuring facilities are adequately staffed and equipped is critical not only for addressing the persistent gaps in access to quality maternal healthcare, but also underscores the need for investments to achieve health equity, as marginalized groups, including poor and ethnic minorities, often bear a disproportionate burden of mortality rates [20,21].

In order to assess the status of public health facilities and establish a baseline for monitoring progress resulting from inputs and technical support provided to the Ministry of Health (MOH), the United States Agency for International Development (USAID)-supported Laos Maternal Child Health and Nutrition (LMCHN) program undertook a comprehensive baseline health facility assessment of maternal and newborn health (MNH) services in its operational area. The World Health Organization’s (WHO) Service Availability and Readiness Assessment (SARA) tool, which is a standardized instrument used to evaluate the capacity of health facilities to provide essential health services, was adapted to capture the structural quality and physical resources, including guidelines, equipment, diagnostics, commodities, and medicines needed for facilities to deliver both general and specific MNH services [22].

The facility survey was one component of a broader suite of baseline assessments, which also involved interviews with healthcare providers and an exploration of the care experiences of users of antenatal care, delivery, and pediatric services. The results presented focus specifically on the availability and readiness of facilities to provide MNH services and will be used to identify opportunities to increase utilization of facility-based information for decision-making about investments in health systems and services in Laos.

## Methods

### Study setting

The study was conducted in the operational area of the LMCHN project, which included 24 districts across the five provinces of Oudomxay and Phongsaly in the north and Savannakhet, Salavan, and Sekong in the south. The study area in Table 1 represents approximately 57% of the provincial districts and 59% of the provincial population. The provinces are 80% rural, relying largely on subsistence farming. Three of the provinces have a population density lower than the national average of 27 people per square kilometer, primarily due to their mountainous terrain. All the provinces have a relatively high multidimensional poverty rate, which measures the percentage of people deprived of three dimensions: monetary poverty, education, and basic infrastructure services [23].

**Table 1:** Characteristics of the assessed provinces.

Province	Population ^A^	Population Density (per square km) ^A^	% Rural ^A^	% Multidimensional poverty headcount rate ^B^^,^^C^
Phongsaly	177,989	11	80.9	23.9
Oudomxay	307,622	20	76.0	34.8
Savannakhet	969,697	45	77.8	33.9
Salavan	396,942	37	88.7	32.9
Sekong	113,048	15	64.8	44.1

^A^ 2015 Lao Statistics Bureau, Population Census;

^B^ 2018/19 Lao Statistics Bureau, Poverty Profile;

^C^ The population with a deprivation score of at least 33.3 percent, expressed as a share of the population.

### Study design and sample

This descriptive, cross-sectional study covered all public facilities at the primary healthcare level in the study area, which included 212 health centers and 20 district hospitals for a total of 232 facilities (S1 Fig). Covering all primary healthcare facilities allowed for a comprehensive set of data to identify gaps in healthcare access, optimize resource allocation, and improve the planning and delivery of services to meet client needs within the project’s operational area. Provincial hospitals were excluded as the project’s mandate focuses on the primary healthcare level.

### Tool adaptation

The study used WHO’s SARA tool, which has been widely used by various organizations to assess health system performance, with wide application across multiple countries and contexts [24]. The tool is designed to evaluate health facilities through three core service domains: 1) service availability, which measures whether there are sufficient facilities and health personnel (doctors, nurses, midwives) available in sufficient quantity relative to the population to support service delivery at the system level; 2) general service readiness, which measures the availability of essential components, such as trained staff, guidelines, equipment, and medicines, needed for the delivery of general health services at the point of care at the facility level; and 3) service specific readiness, which measures the availability of essential components, such as trained staff, guidelines, equipment, and medicines, needed to deliver a specific health service like ANC or MNH, at the point of care at the facility level.

The tool was adapted to focus on essential criteria needed to provide core maternal and newborn health services, including medicines, equipment, and other supplies required per international and local standards. The tool was reviewed by program staff from the MOH’s Department of Healthcare and Rehabilitation (DHR) to ensure it was aligned with MOH standards and pretested at two district hospitals and one health center in Vientiane province prior to implementation.

### Data collection

Eighty-five data enumerators were locally recruited from each province and participated in a five-day training led by trained LMCHN and DHR staff in October 2023. Data collection was completed between October and November 2023. Five data collectors comprised each team, with four teams deployed in Phongsaly and Oudomxay each, and three teams deployed in each of the three remaining provinces in the south.

Data collection immediately followed training and was conducted simultaneously in all five provinces in October and November 2023. On average, the facility survey took between four and six hours depending on the size of the facility, with health centers requiring less time than district hospitals. Data enumerators interviewed the facility in-charge and administrative heads who were most knowledgeable about the specific MNH service areas included in the assessment. Medical equipment was observed to verify its availability and operational status.

Data collection in each province was overseen by one field supervisor and two data managers, who managed the data collection teams, coordinated schedules, and conducted routine quality checks of the collected data. Data were collected on tablets using SurveyCTO, an electronic data collection and management platform [25]. Data entered into tablets in the field were uploaded to a central cloud server, allowing the survey management team to review questionnaires on a daily basis.

### Data management and analysis

Submitted questionnaires were cleaned, coded, and analyzed using Stata SE16 (StataCorp. LLC, College Station, TX) with guidance from the WHO SARA manual. Our analysis focused on service availability, which helps answer whether the health system has the necessary infrastructure and workforce to support service delivery at the population level, and service-specific readiness, which assesses whether individual facilities have the essential inputs, such as trained staff, equipment, and medicines, needed to deliver specific MNH services at the point of care.

Service availability was measured using tracer indicators in two domains: health infrastructure and the health workforce. The health infrastructure domain score was calculated using tracer indicators which included the number of facilities and the number of inpatient and maternity beds, both standardized per 10,000 population. The domain score for the health workforce, which included doctors, nurses, and midwives, was similarly calculated per 10,000 population. The tracer indicators in each domain were expressed as ratios and compared against current WHO-recommended benchmark standards. All indicators for density were calculated using the 2015 Population and Housing Census data, the most recent census data publicly available [26]. A score was then calculated for each domain, representing the percentage of the benchmark has been reached in each province. The mean of both domain scores was then calculated as an overall service availability score.

Tracer indicators needed to measure service readiness for specific maternal and newborn care services (ANC, newborn and delivery care, MNH medicines, and BEmONC readiness) were grouped into three domains: 1) the presence of staff trained in the specific service and the availability of relevant clinical guidelines; 2) the availability of functioning equipment required to provide that service, such as a sphygmomanometer, resuscitation, or sterilization equipment; and 3) the presence of essential medicines and commodities required to provide that service, such as magnesium sulfate, oxytocin, or antibiotics. Each tracer item was coded as binary to reflect whether the item was available (“1”) or not available (“0”) at the facility on the day of the assessment. Medicines and commodities were considered available if at least one item with a valid expiration date was observed in the facility. Functioning equipment and guidelines were considered available if they were observed onsite. Health workers were considered trained if at least one staff member had received a relevant MNH training within the last two years.

Each tracer item was assigned an availability score, representing the proportion of facilities that had the item available on the day of the assessment. The mean of all tracer item availability scores was then calculated as a service-specific readiness score for that particular service area. Each tracer item was given equal weight and only items required by the MOH to be stocked at both district hospitals and health centers were included in the analysis. Results were presented by the two facility levels: health centers and district hospitals. All facilities reported offering the specified MNH service. Therefore, as availability was 100% across all facilities for the specific services, only the readiness results will be presented.

### Ethical considerations

The assessment did not collect any personal, sensitive, or confidential information from human subjects. The study was submitted and approved by both the local review board in Laos the National Ethics Committee for Health Research (NECHR) (ID No. 52, 2023) and JSI’s Institutional Review Board (ID No. 23-48E, 2023). The assessment was also authorized by the MOH’s DHR, which oversees management of all public health facilities in Laos. A formal letter of authorization and cooperation was issued by the DHR to permit entry to the facilities.

Verbal consent was obtained from each respondent prior to any interviews. The consent process was documented directly in the digital survey tool, with the interviewer serving as a witness. The consent form, which outlined the study’s objectives, requirements, potential risks, and privacy rights, emphasizing that the study was voluntary and that they would experience no negative consequences as a result of refusal to participate or drop out, was read to respondents in the Lao language. No minors were recruited or interviewed for the study. All members of the research team were trained in ethical practices and informed consent procedures to ensure compliance with international standards for research involving human subjects as outlined in the 1964 Helsinki Declaration and its later amendments or comparable ethical standards. The process and documentation of verbal consent was approved by both the NECHR and JSI’s IRB.

## Results

Of the 232 healthcare facilities in the study area, 91.4% were health centers and 8.6% were district hospitals, with the majority of all facilities (90.0%) located in a rural area as seen in Table 2. More than a quarter (28.0%) of the facilities were located in Savannakhet. Sekong had the lowest proportion of facilities in the study area, at 14.7%. Only Salavan and Sekong had one district hospital in each of the districts included in the study area with one or more of the remaining districts in Phongsaly, Oudomxay, and Savannakhet lacking a district hospital.

**Table 2:** Distribution of healthcare facilities in study area.

Variable	Frequency (n = 232)	%
**Province**		
Phongsaly	39	16.8
Oudomxay	41	17.7
Savannakhet	65	28.0
Salavan	53	22.8
Sekong	34	14.7
**Location**		
Rural	209	90.0
Urban	23	10.0
**Type of healthcare facility**		
Health center	212	91.4
District hospital	20	8.6

### Service availability

To determine if the health system has the necessary infrastructure and workforce to support service delivery at the population level, service availability was assessed using four tracer indicators across two domains as seen in Table 3. The overall service availability score across all districts in the program area was 71.3%. The average availability score for districts in the northern provinces was approximately seven percentage points higher than the average of districts in the southern provinces, indicating that facilities in the southern provinces may face more availability-related constraints than those in the north.

**Table 3:** Comparison of service availability tracer indicators to WHO target.

		Northern Provinces		Southern Provinces			
Domain and Tracer Indicator	WHO Target	Phongsaly	Oudomxay	Savannakhet	Salavan	Sekong	Total
		N = 5 districts	N = 5 districts	N = 5 districts	N = 5 districts	N = 4 districts	N = 24 districts
Health Services Infrastructure Score (%)	–	100	72.1	56.4	69.1	100	78.7
Facility density (per 10,000 pop.)	2	3.23	1.63	1.76	1.70	3.18	2.00
Inpatient bed density (per 10,000 pop.)	25	16.90	8.62	5.53	10.07	18.22	9.78
Maternity bed density (per 1,000 pregnant pop.)	10	14.15	10.03	5.90	8.22	11.61	9.72
Health Workforce Score (%)	–	78.5	61.2	61.6	55.8	85.7	63.9
Health workforce density (per 10,000 pop.)	23	18.06	14.07	14.16	12.83	19.72	14.70
Mean Service Availability Score							
By province	–	89.3	66.6	59.0	62.5	92.8	71.3
By region	–	77.9		71.4			–

The health services infrastructure and health workforce scores represent the proportion of the WHO target for tracer indicators in each domain that has been reached. Districts in Phongsaly and Sekong provinces achieved 100% of the WHO targets for health services infrastructure, followed by Oudomxay at 72.1%. While Phongsaly, Oudomxay, and Sekong exceed the 10 maternity beds per 1,000 pregnant women, they each fall short of the recommended 25 inpatient beds per 10,000 population. In comparison, the districts in the remaining two provinces of Savannakhet and Salavan did not meet the targets for any of the three indicators within the health services infrastructure domain.

The health workforce score considered all full-time nurses, midwives, and doctors with all degree levels as well as specialized doctors. Both Phongsaly and Sekong scored well, achieving 78.5% and 85.7% of the WHO target of 23 doctors, nurses, and midwives per 10,000 population, respectively. The health workforce score for the remaining provinces was sub-optimal at 61.2%, 61.6%, and 55.8% for Oudomxay, Savannakhet, and Salavan, respectively.

### Antenatal care service readiness

Readiness for antenatal care (ANC) services was assessed using seven tracer items identified in Table 4. The overall ANC mean readiness score across both facility types was 69.0%, CI_95_:62.7–75.3%. This indicates that on average, facilities had about 5 of the 7 ANC tracer items available. District hospitals scored higher than health centers, at 77.1%, CI_95%_:75.4–78.8%, compared to 68.2%, CI_95%_: 62.1–74.3%.

**Table 4:** Percentage of healthcare facilities equipped with tracer items for antenatal care.

Domain Tracer Item	Health Centers (%)	District Hospitals (%)	Total (%)
	N = 212	N = 20	N = 232
Guidelines on ANC	47.6	70.0	49.6
ANC checklists or job aids	46.7	45.0	46.6
Staff trained in ANC	88.2	95.0	88.8
Blood pressure apparatus	93.9	100.0	94.4
Iron tablets	65.1	65.0	65.1
Folic acid tablets	42.9	70.0	45.3
Tetanus toxoid vaccines	92.9	95.0	93.1
Mean ANC Score [CI 95%]	68.2 [62.1-74.3]	77.1 [75.4-78.8]	69.0 [62.7-75.3]

The most notable difference between facility types was in the availability of folic acid tablets and ANC guidelines, with the mean availability of those items being at least 20 percentage points higher among district hospitals than health centers. Blood pressure apparatus and tetanus toxoid vaccine availability was high in both health centers and district hospitals, with mean availability scores for both items exceeding 92% across both facility types. Most (88.8%) of facilities program-wide indicated that they have at least one staff member who has received ANC training within the last two years.

### Newborn and delivery care service readiness

Newborn and delivery care services are provided at both health centers and district hospitals. Readiness was assessed using 23 tracer items encompassing the core equipment, guidelines, medicines, commodities, and staff training needed for a facility to support effective newborn and delivery care services, as identified in Table 5. The overall newborn and delivery mean readiness score was 68.3%, CI_95%_: 59.5–77.1%, indicating that on average, facilities have about 16 of the 23 tracer items available. District hospitals displayed a higher readiness to provide newborn and delivery care services than health centers, scoring 81.3%, CI_95%_:79.7–82.9% compared to 67.0%, CI_95%_:58.2–75.8%.

**Table 5:** Percentage of healthcare facilities equipped with tracer items for newborn and delivery care and BEmONC services.

Domain and Tracer Item	Health Centers (%)	District Hospitals (%)	Total (%)
	N = 212	N = 20	N = 232
**Staff and Guidelines**			
Guidelines on essential childbirth care^*^	27.4	50.0	29.3
Guidelines on essential newborn care^*^	36.8	55.0	38.4
Staff trained in essential childbirth care	59.4	75.0	60.8
Staff trained in newborn resuscitation	58.5	80.0	60.3
Staff trained in BEmONC^*^	–	60.0	–
**Equipment**			
Emergency transport^*^	67.5	95.0	69.8
Sterilization equipment	80.7	95.0	81.9
Examination light	55.7	75.0	57.3
Delivery pack	96.2	100.0	96.6
Manual vacuum extractor^*^	1.9	35.0	4.7
Vacuum aspirator^*^	20.3	75.0	25.0
Neonatal bag and mask^*^	22.6	45.0	24.6
Delivery bed	92.5	90.0	92.2
Partograph	82.5	80.0	82.3
Gloves	95.8	95.0	95.7
Infant weighing scale	75.0	90.0	76.3
Blood pressure apparatus	93.9	100.0	94.4
Soap/running water; alcohol-based rub	98.6	95.0	98.3
**Medicines and Commodities**			
Antibiotic eye ointment for newborn	85.8	85.0	85.8
Injectable uterotonic^*^	92.0	90.0	91.8
Injectable antibiotic^*^	98.6	95.0	98.3
Magnesium sulfate (injectable)^*^	13.2	80.0	19.0
Skin disinfectant^*^	96.2	90.0	95.7
Intravenous solution w/ infusion set^*^	91.0	100.0	91.8
Mean Overall Newborn and Delivery Score [CI 95%]	67.0 [58.2 – 75.8]	81.3 [79.7 – 82.9]	68.3 [59.5 – 77.1]
Mean BEmONC Score [CI 95%]	–	72.5 [62.6 – 82.4]	–

*Tracer item essential to the delivery of BEmONC services at district hospitals.

The largest disparities between facility types were the availability of manual vacuum extractors, injectable magnesium sulfate, vacuum aspirators, and neonatal bags and masks. For example, while 75.0% of district hospitals had a vacuum aspirator available, this was true for only 20.3% of health centers.

Medicines for newborn and delivery care were largely available in the program area, with availability scores of at least 85% for each item. The exception was injectable magnesium sulfate, which was available at just 13.2% of health centers and 19% of facilities program-wide. Equipment availability varied considerably program-wide and between facility types. While gloves, delivery packs, blood pressure apparatuses, and soap and running water or alcohol-based hand rub were available in at least 94% of facilities program-wide, manual vacuum extractors and neonatal bags and masks were available at just 4.7% and 24.6% of facilities, respectively.

Guidelines on essential childbirth care and essential newborn care were rarely available in health centers (27.4% and 36.8%, respectively), but were available in at least half (50.0% and 55.0%, respectively) of district hospitals. More than half (58.5%) of health centers had at least one staff member trained in newborn resuscitation within the two years preceding the assessment. A similar proportion (59.4%) had at least one staff member trained in essential childbirth care in the previous two years. Eighty percent of district hospitals had a staff member trained in newborn resuscitation while 75.0% had a staff member trained in essential childbirth care.

### Life-saving commodities for maternal and newborn health

Of the thirteen commodities identified by the UN Commission as life-saving for women and children, seven are considered essential to health at the newborn and maternal life stages [27]. Those seven commodities, the conditions they treat or prevent, and their availability within health centers and district hospitals is shown in Table 6. With the exception of injectable antibiotics and skin disinfectant, district hospitals were more likely than health centers to have each of the life-saving commodities available.

**Table 6:** Percentage of healthcare facilities equipped with 7 lifesaving commodities for maternal and newborn health according to UN Commission guidance.

Life stage and commodity	Condition	Health Centers (%)	District Hospitals (%)	Total (%)
		N = 212	N = 20	N = 232
**Maternal Health**				
Oxytocin	Post-partum hemorrhage	92.5	100.0	93.1
Misoprostol	Post-partum hemorrhage	2.8	80.0	9.5
Magnesium sulphate	Eclampsia and severe pre-eclampsia	13.2	80.0	19.0
**Newborn Health**				
Injectable antibiotics	Newborn sepsis	98.6	95.0	98.3
Antenatal corticosteroids	Preterm respiratory distress syndrome	38.7	75.0	41.8
Skin disinfectant^*^	Newborn cord care	96.2	90.0	95.7
Resuscitation devices	Newborn asphyxia	53.3	95.0	56.9

*UN Commission guidance identifies chlorhexidine as the life-saving commodity for newborn umbilical cord care. Currently in Laos, betadine and alcohol 70 are used, and chlorhexidine is not. Therefore, this assessment used the broader term “skin disinfectant” as used in the SARA manual.

The availability of essential maternal and newborn health commodities varied across facility types in the program area. Oxytocin, injectable antibiotics, and skin disinfectant were widely available, with each present in at least 90.0% of all facilities. Resuscitation devices, which include a neonatal bag and mask and a suction device, were available at 95% of district hospitals and just over half (53.3%) of health centers. The largest variation between health centers and district hospitals is in the availability of misoprostol, which although was required at both facility types, was available at just 2.8% of health centers compared to 80% of district hospitals. Similarly, despite being available in the majority (80.0%) of district hospitals, just 13.2% of health centers had magnesium sulfate available on the day of the assessment. Antenatal corticosteroids for the prevention of preterm respiratory distress syndrome were available at fewer than half (38.7%) of health centers and three quarters of district hospitals.

### Basic emergency obstetric and newborn care (BEmONC) readiness

Emergency obstetric and newborn care, introduced in 1997, is designed to provide timely treatment for complications to reduce maternal and newborn mortality. A set of seven key obstetric services, or “signal functions,” have been identified as critical to providing BEmONC, including: 1) administration of parenteral antibiotics; 2) administration of parenteral uterotonic drugs; 3) administration of parenteral anticonvulsants; 4) removal of retained products (manual vacuum aspiration); 5) assisted vaginal delivery; 6) manual removal of the placenta; and 7) resuscitation of the newborn [28]. Laos requires BEmONC to be provided at health centers and district hospitals (type A and type B), with comprehensive emergency obstetric and newborn care (CEmONC) required only at type A district hospitals and provincial hospitals. Due to the small number of CEmONC-eligible hospitals in our sample and the exclusion of provincial hospitals from our study, we provide results for BEmONC readiness only.

Service readiness for BEmONC at district hospitals was assessed based on the availability of 12 tracer items essential to the delivery of the seven BEmONC signal functions as seen in Table 5. These tracer items were selected based on their specific use for BEmONC signal function delivery per the SARA manual and their use in previous studies. The mean BEmONC readiness score was 72.5% CI95%: 62.6–82.4, indicating that on average, district hospitals have about 9 of the 12 tracer items available. Manual vacuum extractors were available at just 35% of district hospitals, indicating some constraints on the capacity of these facilities to provide assisted vaginal delivery. More than half (60%) of district hospitals had at least one staff member trained in BEmONC in the two years prior to the assessment.

## Discussion

This cross-sectional analysis provides important insight into the state of MNH and BEmONC service availability and readiness in Laos as well as highlights differentials between provinces and health system levels. The findings identified that the number of public facilities and maternity bed density per population fall short of meeting minimum recommendations for three of the five provinces. Both Phongsaly and Sekong have the lowest population and population densities among the five provinces. However, both provinces are characterized by mountainous terrain with poor infrastructure, with a substantial number of villages located in remote areas that are inaccessible by road for parts of the year.

Coverage indicators such as these are limited as they mask service accessibility as well as other inequities. Previous studies have shown that poorer women, who often live in predominantly rural areas, often receive lower quality care [29]. Alternative measurements for expressing coverage, such as distance required to reach a health facility or route-based distances, would provide a more useful understanding of accessibility from the perspective of the client [30,31].

The study found the availability of health workers to be a significant issue, with all provinces falling short of recommended minimum standards. Previous studies in Laos have also found health workers to be maldistributed across geographies and between health facility levels [32,33]. Such human resource constraints are similar to those in many other countries in the region and beyond as well [34,35]. In addition to an insufficient number of health personnel is the quality of training, limited government allocation to recruit and retain sufficient health personnel, limited management capacity, and incentives for retention in remote areas [36]. However, given current population growth rates in Laos, coupled with the highest adolescent fertility rate in the Southeast Asia region, it will be critical to significantly increase the number of health personnel to meet the demand by 2030 [37].

Several countries have utilized lay health workers in communities to help address shortages in trained facility staff and provide health information and basic health services to those in remote or hard-to-reach areas. Thailand, for example, engages volunteers in providing health education, home visits, basic medical services, disease surveillance, and referrals in villages across the country [38]. Similar programs have been established in a number of other countries [39]. These programs, however, are often highly dependent on donor funding and face a number of challenges in financing, planning, roles and tasks, recruitment and training, and incentives among other issues [40].

Laos has a Village Health Volunteer (VHVs) program managed under the Ministry of Health (MOH). As a substantial proportion of the population that live in remote areas with poor infrastructure, often populated by ethnic minorities creating additional cultural and linguistic barriers with health workers, VHVs have the potential to fill an existing gap and expand the reach of the formal health system [41]. However, there is a lack of financial, training, and other resources available to fully support this approach [42]. Additionally, VHVs are often political appointments selected by village leaders, and a gender imbalance favors male VHVs [43]. While some VHVs have been trained in early diagnosis and treatment strategies for malaria and support for postnatal depressive symptoms, these programs are not widespread nor continuous, and VHVs are generally limited in terms of their mandate and scope of activities allowed [44,45].

Ensuring the availability, affordability, and quality of essential commodities is vital for reducing maternal deaths, but our findings showed that access to several essential life-saving medications continues to be a significant challenge for both ANC and MNH services [46]. While it is difficult to draw comparisons with other countries due to wide variation in systems and health system support, a similar lack of available life-saving medicines has been found in other countries that struggle to achieve optimal readiness. For example, the availability of seven essential maternal and reproductive health medicines in Myanmar was found to be only 52.9% [47]. Additionally, a review in 12 countries in sub-Saharan Africa found significant stockouts of oxytocin and shortages of magnesium sulfate [48].

Our study found significant shortages in several commodities, including iron and folic acid, magnesium sulfate, misoprostol, and antenatal corticosteroids. Yet these medicines can help address a number of adverse pregnancy and neonatal conditions and outcomes. Iron and folic acid, for example, provide critical protection for the growth and neurodevelopment of the fetus as well as other against other adverse pregnancy and infant outcomes [49,50]. Magnesium sulfate is a commonly used medication that can prevent eclamptic seizures and provide neuroprotection for the fetus [51]. The lack of medicines directly undermines maternal health service readiness and directly impacts the ability to manage common pregnancy-related complications.

The shortage of essential medicines may be attributed to factors such as higher demand, insufficient funding for medicine supplies, an overreliance on external funding, or inefficient logistics and supply chain management systems [52,53]. At the time of publication, for example, both iron and folic acid have been stocked out nationwide in Laos for almost a year. Ensuring a dependable supply of essential medicines must be a high priority so that healthcare providers are able to offer timely and effective interventions without putting both the mother and baby at significant risk. Additionally, the absence of medications can lead to increased reliance on less effective or outdated treatments, contributing to higher maternal and neonatal mortality rates. Ultimately, inadequate access to essential medicines erodes the quality of maternal care, making it much harder to achieve positive health outcomes and meet global maternal health targets.

There was also a lack of guidelines available for both ANC and MNH services, especially in health centers compared to district hospitals. Clinical guidelines are an important quality standard that can help optimize patient care by ensuring common standards and practices of care are implemented and manage risk [54]. Previous studies have shown that guidelines can improve the quality and consistency of care received [55,56]. However, it is also important to recognize that availability of guidelines cannot fully address the know-do gap, or the disconnect between translating what is known to work into the care patients receive, which can lead to poor quality of care [57,58]. Additionally, a previous study in Laos also found inadequate awareness and use of clinical guidelines even when they are available [59]. Improving use of guidelines is equally important as increasing availability.

The findings also identified a lack of some basic equipment such as examination lights, manual vacuum extractors, vacuum aspirators, neonatal bag and masks, and infant weighing scales. Previous studies have linked a lack of basic examination equipment to poor facility performance and low utilization of ANC, which is an important gateway to continued use of services throughout pregnancy, delivery, and the postnatal period [60,61]. Additionally, being able to provide adequate care during ANC has been shown to decrease adverse pregnancy outcomes and related morbidities and mortalities through the early detection and treatment of medical or other obstetric complications and improved outcomes [62].

The findings also revealed that in general district hospitals are better equipped compared to health centers. Service readiness for ANC was 69.0% at health centers compared to 77.1% at district hospitals. A wider gap existed for newborn and delivery care, with service readiness at 67.0% for health centers compared to 81.3% for district hospitals. This is consistent with findings from Laos and other countries, which suggest that health centers are frequently bypassed in favor of district hospitals, where there is a perception of better equipment, more comprehensive services, and higher quality of care [62]. Additionally, the health system in Laos does not mandate referrals for accessing care at higher levels, further contributing to the preference for district hospitals [63]. This represents a substantial gap in the continuity of care as well as early detection of pregnancy complications. It may also lead to fewer ANC contacts, as district hospitals are farther from communities than health centers.

Inadequacy of facilities to provide BEmONC services limits accessibility of critical services that can narrow disparities in maternal and newborn deaths, especially in rural areas that already carry a disproportionate burden of the mortality rates [64]. Where required, district hospitals lack several of the tracer items. Previous studies have shown that low availability of BEmONC services is associated with a higher burden of maternal and newborn deaths [1]. This also represents a substantial obstacle to universal access to health services. This is exacerbated by the lack of a consistent supply of medicines and available equipment to provide basic preventive and treatment services for maternal and newborn health.

Based on the findings of this analysis, several key recommendations emerge to guide efforts to strengthen MNH service availability and readiness in Laos, particularly in underserved and remote areas. Policymakers should prioritize expanding public health infrastructure by increasing the number of public facilities and maternity beds proportionate to population demands. Concurrently, implementation of mobile outreach services is recommended to address geographic and seasonal barriers to care. Additionally, fostering strategic public-private partnerships can leverage private sector capacity to support infrastructure development, supply chain management, and mobile service delivery.

To mitigate the persistent shortage and maldistribution of skilled MNH personnel across provinces, targeted interventions must prioritize the recruitment, training, and retention of qualified health workers in rural and remote areas. Effective retention strategies should encompass comprehensive financial incentives, improved working conditions, and sustained opportunities for professional development. These actions are critical in light of Laos’s demographic trajectory characterized by population growth and a high adolescent fertility rate, which collectively forecast increased demand for MNH services in the near future.

Addressing shortages of essential MNH equipment and medicines necessitates a strategic focus on strengthening supply chain systems. Policymakers should institutionalize regular logistics assessments to detect and resolve inefficiencies, improve demand forecasting, and streamline procurement and distribution processes. Equally important is building the capacity of health facility staff in inventory management and leveraging digital tools to maintain consistent availability of critical commodities and functional equipment.

While the study provides an objective assessment of the capacity of health centers and district hospitals in the study area, there are several limitations. First, it was restricted to the operational area of the project. Although the assessment covered all health centers and district hospitals within the operational area, representing 24 of the 41 districts (58.5%) and 59% of the population, the results are not reflective of the province as a whole. Second, other factors related to facility size and geographic access to services were not covered in this assessment but are equally important considerations in increasing coverage and use of services.

Third, a limited number of tracer items was used and only included those required in both health center and district hospitals. While this provides an important snapshot of the critical gaps, it does not provide a complete picture of the extent to which minimum service standards are met at each facility level. Finally, the dimensions of quality in terms of provider skills and service delivery were not assessed, although aspects of these were covered in additional components of the full assessment. Previous studies have linked a lack of equipment and supplies to deteriorating health worker skills, which can affect the quality of care received [65,66].

## Conclusion

The findings reveal significant gaps in the availability of essential items for maternal and newborn health services in public facilities across Laos, with the greatest challenges seen at the health center level. These gaps present a powerful opportunity for transformation. Strategic investments in critical areas—such as staffing, infrastructure, equipment, and medicines—are not only urgently needed but also hold the potential to dramatically improve health outcomes and empower health personnel with the tools they need to succeed. This baseline serves as a rallying call for policymakers and stakeholders to prioritize impactful interventions. By strengthening supply chains, increasing domestic health financing, upgrading infrastructure, and advancing workforce recruitment and retention strategies, we can increase access to healthcare services for all. Targeted policies, bold investments, and dynamic partnerships will be the keys to ensuring every community in Laos can access the quality healthcare services they need.

## Supporting information

S1 Table Characteristics of the assessed provinces. (DOCX)

S2 Table Distribution of healthcare facilities in study area. (DOCX)

S3 Table Comparison of service availability tracer indicators to WHO target. (DOCX)

S4 Table Percentage of healthcare facilities equipped with tracer items for antenatal care. (DOCX)

S5 Table Percentage of healthcare facilities equipped with tracer items for newborn and delivery care and BEmONC services. (DOCX)

S6 Table Percentage of healthcare facilities equipped with 7 lifesaving commodities for maternal and newborn health according to UN Commission guidance. (DOCX)

S1 Fig Geographic distribution of the assessed health facilities across the five provinces. (TIF)

S1 Data Database and Questionnaire. (ZIP)
