# Supplementary material for: Assessing maternal and newborn health readiness: Insights from a service availability assessment in five provinces in Laos
Source: PLoS One. 2025 Sep 11;20(9):e0331659. doi: 10.1371/journal.pone.0331659 (PMC12425213; doi:10.1371/journal.pone.0331659)
Supplement: S1 Table — (DOCX) [file pone.0331659.s001.docx]

**Table 1. Characteristics of the assessed provinces**

| Province | Population ^A^ | Population Density (per square km) ^A^ | % Rural ^A^ | % Multidimensional poverty headcount rate ^B C^ |
| --- | --- | --- | --- | --- |
| Phongsaly | 177,989 | 11 | 80.9 | 23.9 |
| Oudomxay | 307,622 | 20 | 76.0 | 34.8 |
| Savannakhet | 969,697 | 45 | 77.8 | 33.9 |
| Salavan | 396,942 | 37 | 88.7 | 32.9 |
| Sekong | 113,048 | 15 | 64.8 | 44.1 |
| ^A^ 2015 Lao Statistics Bureau, Population Census; ^B^ 2018/19 Lao Statistics Bureau, Poverty Profile; ^C^ The population with a deprivation score of at least 33.3 percent, expressed as a share of the population. | | | | |
