# Supplementary material for: Assessing maternal and newborn health readiness: Insights from a service availability assessment in five provinces in Laos
Source: PLoS One. 2025 Sep 11;20(9):e0331659. doi: 10.1371/journal.pone.0331659 (PMC12425213; doi:10.1371/journal.pone.0331659)
Supplement: S2 Table — (DOCX) [file pone.0331659.s002.docx]

**Table 2. Distribution of healthcare facilities in study area**

| Variable | Frequency (n=232) | % |
| --- | --- | --- |
| **Province** |  |  |
| Phongsaly | 39 | 16.8 |
| Oudomxay | 41 | 17.7 |
| Savannakhet | 65 | 28.0 |
| Salavan | 53 | 22.8 |
| Sekong | 34 | 14.7 |
| **Location** |  |  |
| Rural | 209 | 90.0 |
| Urban | 23 | 10.0 |
| **Type of healthcare facility** |  |  |
| Health center | 212 | 91.4 |
| District hospital | 20 | 8.6 |
