# Supplementary material for: Assessing maternal and newborn health readiness: Insights from a service availability assessment in five provinces in Laos
Source: PLoS One. 2025 Sep 11;20(9):e0331659. doi: 10.1371/journal.pone.0331659 (PMC12425213; doi:10.1371/journal.pone.0331659)
Supplement: S3 Table — (DOCX) [file pone.0331659.s003.docx]

**Table 3. Comparison of service availability tracer indicators to WHO target**

|  |  | Northern Provinces | | Southern Provinces | | |  |
| --- | --- | --- | --- | --- | --- | --- | --- |
| Domain and Tracer Indicator | WHO Target | Phongsaly | Oudomxay | Savannakhet | Salavan | Sekong | Total |
|  |  | N= 5 districts | N=5 districts | N=5  districts | N=5 districts | N=4 districts | N=24 districts |
| Health Services Infrastructure Score (%) | - | 100 | 72.1 | 56.4 | 69.1 | 100 | 78.7 |
| Facility density  (per 10,000 pop.) | 2 | 3.23 | 1.63 | 1.76 | 1.70 | 3.18 | 2.00 |
| Inpatient bed density  (per 10,000 pop.) | 25 | 16.90 | 8.62 | 5.53 | 10.07 | 18.22 | 9.78 |
| Maternity bed density  (per 1,000  pregnant pop.) | 10 | 14.15 | 10.03 | 5.90 | 8.22 | 11.61 | 9.72 |
| Health Workforce Score (%) | - | 78.5 | 61.2 | 61.6 | 55.8 | 85.7 | 63.9 |
| Health workforce density  (per 10,000 pop.) | 23 | 18.06 | 14.07 | 14.16 | 12.83 | 19.72 | 14.70 |
| Mean Service Availability Score | | | | | | | |
| By province | - | 89.3 | 66.6 | 59.0 | 62.5 | 92.8 | 71.3 |
| By region | - | 77.9 | | 71.4 | | | - |
