# Supplementary material for: Assessing maternal and newborn health readiness: Insights from a service availability assessment in five provinces in Laos
Source: PLoS One. 2025 Sep 11;20(9):e0331659. doi: 10.1371/journal.pone.0331659 (PMC12425213; doi:10.1371/journal.pone.0331659)
Supplement: S4 Table — (DOCX) [file pone.0331659.s004.docx]

**Table 4. Percentage of healthcare facilities equipped with tracer items for antenatal care**

| Domain Tracer Item | Health Centers (%) | District Hospitals (%) | Total (%) |
| --- | --- | --- | --- |
|  | N=212 | N=20 | N=232 |
| Guidelines on ANC | 47.6 | 70.0 | 49.6 |
| ANC checklists or job aids | 46.7 | 45.0 | 46.6 |
| Staff trained in ANC | 88.2 | 95.0 | 88.8 |
| Blood pressure apparatus | 93.9 | 100.0 | 94.4 |
| Iron tablets | 65.1 | 65.0 | 65.1 |
| Folic acid tablets | 42.9 | 70.0 | 45.3 |
| Tetanus toxoid vaccines | 92.9 | 95.0 | 93.1 |
| Mean ANC Score  [CI 95%] | 68.2 [62.1-74.3] | 77.1 [75.4-78.8] | 69.0 [62.7-75.3] |
