# Supplementary material for: Assessing maternal and newborn health readiness: Insights from a service availability assessment in five provinces in Laos
Source: PLoS One. 2025 Sep 11;20(9):e0331659. doi: 10.1371/journal.pone.0331659 (PMC12425213; doi:10.1371/journal.pone.0331659)
Supplement: S5 Table — (DOCX) [file pone.0331659.s005.docx]

**Table 5. Percentage of healthcare facilities equipped with tracer items for newborn and delivery care and BEmONC services**

| Domain and Tracer Item | Health Centers (%) | District Hospitals (%) | Total (%) |
| --- | --- | --- | --- |
|  | N=212 | N=20 | N=232 |
| **Staff and Guidelines** |  |  |  |
| Guidelines on essential childbirth care* | 27.4 | 50.0 | 29.3 |
| Guidelines on essential newborn care* | 36.8 | 55.0 | 38.4 |
| Staff trained in essential childbirth care | 59.4 | 75.0 | 60.8 |
| Staff trained in newborn resuscitation | 58.5 | 80.0 | 60.3 |
| Staff trained in BEmONC* | - | 60.0 | - |
| **Equipment** |  |  |  |
| Emergency transport* | 67.5 | 95.0 | 69.8 |
| Sterilization equipment | 80.7 | 95.0 | 81.9 |
| Examination light | 55.7 | 75.0 | 57.3 |
| Delivery pack | 96.2 | 100.0 | 96.6 |
| Manual vacuum extractor* | 1.9 | 35.0 | 4.7 |
| Vacuum aspirator* | 20.3 | 75.0 | 25.0 |
| Neonatal bag and mask* | 22.6 | 45.0 | 24.6 |
| Delivery bed | 92.5 | 90.0 | 92.2 |
| Partograph | 82.5 | 80.0 | 82.3 |
| Gloves | 95.8 | 95.0 | 95.7 |
| Infant weighing scale | 75.0 | 90.0 | 76.3 |
| Blood pressure apparatus | 93.9 | 100.0 | 94.4 |
| Soap/running water; alcohol-based rub | 98.6 | 95.0 | 98.3 |
| **Medicines and Commodities** |  |  |  |
| Antibiotic eye ointment for newborn | 85.8 | 85.0 | 85.8 |
| Injectable uterotonic* | 92.0 | 90.0 | 91.8 |
| Injectable antibiotic* | 98.6 | 95.0 | 98.3 |
| Magnesium sulfate (injectable)* | 13.2 | 80.0 | 19.0 |
| Skin disinfectant* | 96.2 | 90.0 | 95.7 |
| Intravenous solution w/ infusion set* | 91.0 | 100.0 | 91.8 |
| Mean Overall Newborn and Delivery Score [CI 95%] | 67.0  [58.2 – 75.8] | 81.3  [79.7 – 82.9] | 68.3  [59.5 – 77.1] |
| Mean BEmONC Score  [CI 95%] | - | 72.5  [62.6 – 82.4] | - |
| *****Tracer item essential to the delivery of BEmONC services at district hospitals. | | | |
