# Supplementary material for: Assessing maternal and newborn health readiness: Insights from a service availability assessment in five provinces in Laos
Source: PLoS One. 2025 Sep 11;20(9):e0331659. doi: 10.1371/journal.pone.0331659 (PMC12425213; doi:10.1371/journal.pone.0331659)
Supplement: S6 Table — (DOCX) [file pone.0331659.s006.docx]

**Table 6. Percentage of healthcare facilities equipped with 7 lifesaving commodities for maternal and newborn health according to UN Commission guidance**

| Life stage and commodity | Condition | Health Centers (%) | District Hospitals (%) | Total (%) |
| --- | --- | --- | --- | --- |
|  |  | N=212 | N=20 | N=232 |
| **Maternal Health** |  |  |  |  |
| Oxytocin | Post-partum hemorrhage | 92.5 | 100.0 | 93.1 |
| Misoprostol | Post-partum hemorrhage | 2.8 | 80.0 | 9.5 |
| Magnesium sulphate | Eclampsia and severe pre-eclampsia | 13.2 | 80.0 | 19.0 |
| **Newborn Health** |  |  |  |  |
| Injectable antibiotics | Newborn sepsis | 98.6 | 95.0 | 98.3 |
| Antenatal corticosteroids | Preterm respiratory distress syndrome | 38.7 | 75.0 | 41.8 |
| Skin disinfectant* | Newborn cord care | 96.2 | 90.0 | 95.7 |
| Resuscitation devices | Newborn asphyxia | 53.3 | 95.0 | 56.9 |
| *UN Commission guidance identifies chlorhexidine as the life-saving commodity for newborn umbilical cord care. Currently in Laos, betadine and alcohol 70 are used, and chlorhexidine is not. Therefore, this assessment used the broader term “skin disinfectant” as used in the SARA manual. | | | | |
