# Supplementary material for: Assessing maternal and newborn health readiness: Insights from a service availability assessment in five provinces in Laos
Source: PLoS One. 2025 Sep 11;20(9):e0331659. doi: 10.1371/journal.pone.0331659 (PMC12425213; doi:10.1371/journal.pone.0331659)
Supplement: S1 Data — (ZIP) [file pone.0331659.s008.zip › Database and Questionnaire/Health Facility Assessment Tool.docx]

# TOOL 1: FACILITY TOOL^[[1]](#footnote-1)^

| ^[[2]](#footnote-2)^No. | Question | Response | Skip |
| --- | --- | --- | --- |
| **MODULE 1: GENERAL INFORMATION AND SERVICE AVAILABILITY** | | | |
| **SECTION 1: FACILITY IDENTIFICATION** | | | |
| INTERVIEWER INSTRUCTIONS: For Module 1, ask to speak to the facility-in-charge or similar position who can answer general questions about the facility infrastructure, staffing, and management. | | | |
| **104** | Type of Facility | Health Center-A 1  Health Center-B 2  District Hospital-A 3  District Hospital-B 4 |  |
| **106** | Province | Phongsaly 1  Oudomxay 2  Sekong 3  Savannakhet 4  Salavan 5 |  |
| **107** | District | **Phongsaly**  Phongsaly 1  Mai 2  Somphan 3  Bounnua 4  Bountay 5  **Oudomxay**  Xay 6  Nga 7  Beng 8  Houn 9  Pakbeng 10  **Sekong**  Lamam 11  Kalum 12  Dakchung 13  Thateng 14  **Savannakhet**  Atsaphone 15  Songkhone 16  Phalanaxay 17  Xaibuli 18  Champhone 19  **Saravane**  Saravane 20  Lakhonepheng 21  Toumlane 22  Khonepheng 23  Lao ngarm 24 |  |
| **109** | Urban/Rural | Urban (zone 1) 1  Rural with road (zone 2) 2  Rural without road (zone 3) 3 |  |
| **SECTION 2: FACILITY BEDS** | | | |
| **201** | Does this facility have any beds for adults and children (for any purpose – overnight, inpatient, observation, or delivery services)? | No 0  Yes 1 | If NO, SKIP to Q301 |
| **202** | How many overnight / inpatient / observation beds in total does this facility have, both for adults and children (excluding any delivery beds)? (2 digits)  *These are beds for someone receiving care at the health facility or staying overnight. It excludes delivery beds.*  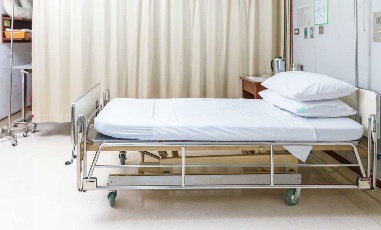 | Two-digits  If NONE, use ‘00’ |  |
| **203** | How many delivery beds in total does this facility have? (2 digits)  *A delivery bed is a specially designed bed where a mother lies to give birth.*  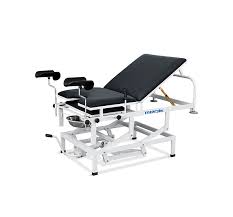 | Two-digits  If NONE, use ‘00’ |  |
| **SECTION 3: STAFFING** | | | |
| I have a few questions on staffing for this facility. Please tell me how many staff with each of the following qualifications are currently assigned to, employed by, or seconded to this facility. Please count each staff member only once, on the basis of the highest technical or professional qualification. I would also like to know, of the total number, how many are part-time in this facility. | | | |
|  | How many [*insert position title*] are there in this facility? | **A**  Number full-time employed including volunteers | **B**  Of those in A, the number volunteer staff. If NONE, enter “0”. |
| **301** | Nurse - Associate Degree |  |  |
| **302** | Nurse – Diploma Level |  |  |
| **303** | Nurse – Higher Diploma Level |  |  |
| **304** | Nurse - Bachelor Degree |  |  |
| **305** | Midwife – Associate Degree |  |  |
| **306** | Midwife – Diploma Level |  |  |
| **307** | Midwife - Higher Diploma Level |  |  |
| **308** | Midwife – Bachelor Degree |  |  |
| **309** | Medical Doctor (General) – Associate Degree |  |  |
| **310** | Medical Doctor (General) – Diploma Level |  |  |
| **311** | Medical Doctor (General)– Higher Diploma Level |  |  |
| **312** | Medical Doctor (General) – Bachelor Degree |  |  |
| **313** | Medical Doctor (General) – Master Degree |  |  |
| **314** | Medical Doctor Specialized (Obstetrics) - Level 1 |  |  |
| **315** | Medical Doctor Specialized (Pediatrics) – Level 1 |  |  |
| **316** | Medical Doctor Specialized (Surgery) – Level 1 |  |  |
| **317** | Medical Doctor Specialized (Internal Medicine) – Level 1 |  |  |
| **318** | Medical Doctor Specialized (Obstetrics) - Level 2 |  |  |
| **319** | Medical Doctor Specialized (Pediatrics) – Level 2 |  |  |
| **320** | Medical Doctor Specialized (Surgery) – Level 2 |  |  |
| **321** | Medical Doctor Specialized (Internal Medicine) – Level 2 |  |  |
| **322** | Laboratorist – Associate Degree |  |  |
| **323** | Laboratorist – Diploma Level |  |  |
| **324** | Laboratorist – Higher Diploma Level |  |  |
| **325** | Laboratorist – Bachelor Degree |  |  |
| **326** | Pharmacist – Associate Degree |  |  |
| **327** | Pharmacist – Diploma Level |  |  |
| **328** | Pharmacist –Higher Diploma Level |  |  |
| **329** | Pharmacist – Bachelor Degree |  |  |
| **330** | Dentist - Associate Degree |  |  |
| **331** | Dentist - Diploma Level |  |  |
| **332** | Dentist - Higher Diploma Level |  |  |
| **333** | Dentist - Bachelor |  |  |
| **334** | Anesthesiologist - Associate Degree |  |  |
| **335** | Anesthesiologist - Diploma Level |  |  |
| **336** | Anesthesiologist - Higher Diploma Level |  |  |
| **337** | Anesthesiologist - Bachelor |  |  |
| **338** | Radiologist and ultrasound technician - Associate Degree |  |  |
| **339** | Radiologist and ultrasound technician - Diploma Level |  |  |
| **340** | Radiologist and ultrasound technician - Higher Diploma Level |  |  |
| **341** | Radiologist and ultrasound technician - Bachelor |  |  |
| **342** | Primary Health Care / Hygiene (Assistant) – Associate |  |  |
| **343** | Environment Hygiene (Cleaning Staff) |  |  |
| **344** | Other (medical or administrative) |  |  |

| **MODULE 2: GENERAL SERVICE READINESS** | | | |
| --- | --- | --- | --- |
| **SECTION 7: INFRASTRUCTURE** | | | |
| **COMMUNICATIONS** | | | |
| **701** | Does this facility have a **functioning landline telephone** that is available to call outside at all times client services are offered?  **CLARIFY THAT IF FACILITY OFFERS 24-HOUR EMERGENCY SERVICES, THEN THIS REFERS TO 24-HOUR AVAILABILITY.** | No 0  Yes 1 |  |
| **702** | Does this facility have a **functioning cellular telephone** with the cost supported by the facility? | No 0  Yes 1 |  |
| **703** | Does this facility have access to a **privately owned cellular telephone** with the cost supported privately (not supported by the facility)? | No 0  Yes 1 |  |
| **704** | Does this facility have a **functioning short-wave radio** for radio calls? | No 0  Yes 1 |  |
| **705** | Does this facility have a **functioning laptop, desktop computer, or tablet**? | No 0  Yes 1 |  |
| **706** | Is there access to email or internet via computer, tablet, mobile phone, or any other device within the facility? | No 0  Yes 1 |  |
| **AMBULANCE/TRANSPORT FOR EMERGENCIES** | | | |
| **707** | Does this facility have a **functional ambulance or any other vehicle for emergency transportatio**n that is regularly stationed at this facility OR operates from this facility? | No 0  Yes 1 | If YES, SKIP to Q709 |
| **708** | Does this facility **have access to** an ambulance or other vehicle for emergency transport for clients that is stationed at or operates from another facility in near proximity? | No 0  Yes 1  DK 98 | ALL RESPONSES, SKIP to Q710 |
| **709** | Is there fuel for the ambulance or other emergency vehicle available today? | No 0  Yes 1  DK 98 |  |
| **POWER SUPPLY** | | | |
| **710** | Does your facility have electricity from any source (e.g., electrical grid, generator, solar, water turbine, or other)? | No 0  Yes 1 | If NO, SKIP to Q720 |
| **711** | What is the **PRIMARY** source of electricity of this health facility?  SELECT ONE | Electric Grid 1  Generator (fuel or battery operated) 2  Solar 3  Water turbine 4  Other (specify) 99 |  |
| **712** | What is the electricity used for in this facility?  SELECT ONE | All electrical needs of the facility 1  Lighting, communications, and some of the medical devices / appliances 2  Only lighting and communications 3  Only medical devices / appliances (e.g., EPI cold room, refrigerator, suction apparatus, etc.) 4 |  |
| **713** | Other than the primary source of electricity, does the facility have any additional or back-up sources of electricity in this facility? | No 0  Yes 1 | If NO, SKIP to Q715 |
| **714** | What is the **SECONDARY or BACK-UP** source of electricity of this health facility?  SELECT ONE | Electric Grid 1  Generator (fuel or battery operated) 2  Solar 3  Water turbine 4  Other (specify) 99 |  |
| **715** | During the past 7 days, was electricity available from the primary OR secondary electrical sources when the facility was open for services? | No 0  Yes 1 | If NO, SKIP to Q720 |
| **716** | How often was electricity available from the primary or secondary source? | Always available (no interruptions) 1  Often available (interruptions of less than 2 hours per day) 2  Sometimes available (frequent or prolonged interruptions of more than 2 hours per day) 3  DK 98 |  |
|  | *FOR INTERVIEWER ONLY (OR PROGRAMMING):*  *Did the respondent indicate that they used a generator as primary or secondary power source?*  *Q711 = 2*  *Q714 = 2* | No 0  Yes 1 | If NO, SKIP to Q720 |
| **717** | Is the generator functional? | No 0  Yes 1  DK 98 |  |
| **718** | Is there fuel or a charged battery available for the generator today? | No 0  Yes 1  DK 98 |  |
|  | *FOR INTERVIEWER ONLY (OR PROGRAMMING):*  *Did the respondent indicate that they used a solar system as primary or secondary power source?*  *Q711 = 3*  *Q714 = 3* | No 0  Yes 1 | If NO, SKIP to Q720 |
| **719** | Is the solar system functional? | No 0  Yes 1  DK 98 |  |
| **BASIC CLIENT AMENITIES** | | | |
| **720** | How many days per week is this facility open? | open; one digit; responses possible: 1-7 for number of days) |  |
| **721** | On average, how many hours per day is this facility open?  SELECT ONE | 4 hours or less 1  5 - 8 hours 2  9 - 16 hours 3  17 - 23 hours 4  24 hours 5 |  |
| **722** | What is the **most commonly** used source of water for this facility at this time?  SELECT ONE | No water source 0  Piped into the facility 1  Piped onto facility grounds 2  Public Tap outside facility grounds 3  Tubewell / borehole 4  Protected dug well 5  Unprotected dug well 6  Protected spring 7  Unprotected spring 8  Rainwater collection 9  Bottled water 10  Cart with small tank / drum 11  Tanker truck 12  Surface water 13  DK 98  Other (specify) 99 | If NO, SKIP to Q724 |
| **723** | Is water available from this source today? | No 0  Yes 1  DK 98 |  |
| **724** | Is there a room (such as a privacy room or a separate consultation room) with auditory and visual privacy available for patient consultation in this facility?  SELECT ONE | Auditory privacy only (a room where others cannot hear them but may see them) 1  Visual privacy only (a room where others cannot see them but may hear them) 2  Both auditory and visual privacy (a room where others cannot see or hear them) 3  No privacy 4 |  |
| **725** | Is there a toilet or latrine on the premises in **functioning condition** that is accessible to the general outpatient client use? | No 0  Yes 1 | If NO, SKIP to Q727 |
| **726** | What type of toilet is available on the premises? Consider the most modern type available.  SELECT ONE | No toilet facility/latrine on site 0  Flush toilet 1  Ventilated Improved Pit Latrine (VIP) 2  Other (specify) 3 |  |
| **INFECTION CONTROL** | | | |
| **727** | Does this facility have the *Infection Prevention and Control Guideline for Primary and Secondary Level*?  IF YES, ASK TO SEE THE DOCUMENT. | No 0  Yes, available and observed 1  Yes, reported available but not observed 2 |  |
| **728** | Does this facility have the *Guideline for the Sterilization and Disinfection for Medical Equipment*? | No 0  Yes, available and observed 1  Yes, reported available but not observed 2 |  |
| Please tell me if the following items used for processing of equipment for reuse are available and functional in the facility today. **IF AVAILABLE, ASK TO SEE IT AND INDICATE IF IT IS FUNCTIONING OR NOT.** | | | |
| **729a** | Does this facility have an electric autoclave (pressure and wet heat)?  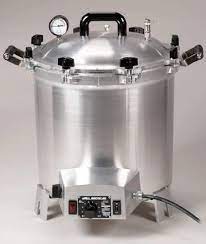 | Not required 0  REQUIRED  Required but not available 1  Yes, observed 2  Yes, reported but not seen 3 | If NO/NA, SKIP to Q730a |
| **729b** | Is this electric autoclave (pressure and wet heat) functional today? | No 0  Yes 1  DK 98 |  |
| **730a** | Does this facility have a non-electric autoclave?  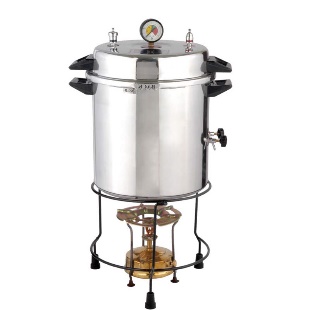 | Not required 0  REQUIRED  Required but not available 1  Yes, observed 2  Yes, reported but not seen 3 | If NO/NA, SKIP to Q731a |
| **730b** | Is this non-electric autoclave functional today? | No 0  Yes 1  DK 98 |  |
| **731a** | Does this facility have an electric dry heat sterilizer?  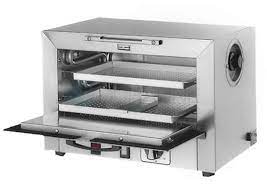 | Not required 0  REQUIRED  Required but not available 1  Yes, observed 2  Yes, reported but not seen 3 | If NO/NA, SKIP to Q732a |
| **731b** | Is this electric dry heat sterilizer functional today? | No 0  Yes 1  DK 98 |  |
| **732a** | Does this facility have an electric boiler or steamer (no pressure)?  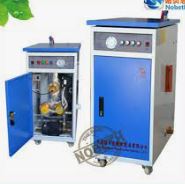 | Not required 0  REQUIRED  Required but not available 1  Yes, observed 2  Yes, reported but not seen 3 | If NO/NA, SKIP to Q733a |
| **732b** | Is this electric boiler or streamer (no pressure) functional today? | No 0  Yes 1  DK 98 |  |
| **INFECTION PREVENTION PRECAUTIONS** | | | |
| Please tell me if the following resources/supplies used for infection control are available in the general outpatient area of this facility today.  **ASK TO SEE THE ITEMS** | | | |
| **733a** | Clean running water (piped, bucket with tap, or pour pitcher) | Not available 0  Yes, observed 1  Yes, reported but not seen 2 |  |
| **733b** | Hand-washing soap/liquid soap | Not available 0  Yes, observed 1  Yes, reported but not seen 2 |  |
| **733c** | Alcohol based hand rub | Not available 0  Yes, observed 1  Yes, reported but not seen 2 |  |
| **733d** | Disposable latex gloves | Not available 0  Yes, observed 1  Yes, reported but not seen 2 |  |
| **733e** | Waste basket (pedal bin) with lid and plastic bin liner | Not available 0  Yes, observed 1  Yes, reported but not seen 2 |  |
| **733f** | Hygiene cleaner or disinfectant (floor or counter cleaning agent such as chlorine)  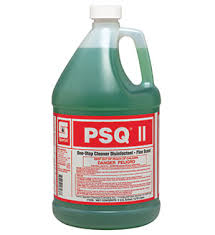 | Not available 0  Yes, observed 1  Yes, reported but not seen 2 |  |
| **733g** | Disposable syringes with disposable needles (used once).  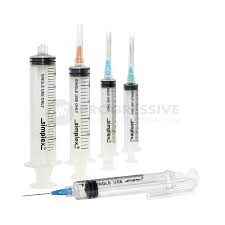 | Not available 0  Yes, observed 1  Yes, reported but not seen 2 |  |
| **733h** | Auto-disable syringes (needles that automatically disable and lock after one use).  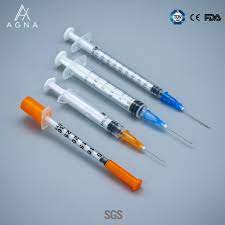 | Not available 0  Yes, observed 1  Yes, reported but not seen 2 |  |
| **HEALTH CARE WASTE MANAGEMENT** | | | |
| Now I would like to ask you a few questions about waste management practices for sharps waste, such as needles or blades.  **PROBE AS NEEDED. IF ANY OF THE RSPONSES 1-8 TAKE PLACE OUTSIDE THE FACILITY, THE CORRECT RESPONSE IS “REMOVE OFFSITE”.** | | | |
| **734** | How does this facility **finally** dispose of sharps waste (e.g., filled sharps boxes)?  *By sharps waste I mean any device or object used to puncture or cut the skin (such as scalpels, needles, etc.)* | Never has sharp waste 0  **BURN INCINERATOR**  2-chamber industrial (800-1000+°C) 1  1-chamber drum/brick 2  **OPEN BURNING**  Flat ground – no protection 3  Pit or protected ground 4  **DUMP WITHOUT BURNING**  Flat ground – no protection 5  Covered pit or pit latrine 6  Open pit – no protection 7  Protected ground or pit 8  **REMOVE OFFSITE**  Stored in a covered container 9  Stored in other protected environment 10  Stored unprotected 12  Other (specify) 99 |  |
| Now I would like to ask you a few questions about waste management practices for medical waste other than sharps, such as used bandages.  **PROBE AS NEEDED. IF ANY OF THE RESPONSES 1-8 TAKE PLACE OUTSIDE THE FACILITY, THE CORRECT RESPONSE IS “REMOVE OFFSITE”.** | | | |
| **735** | How does this facility **finally** dispose of medical waste other than sharps boxes?  *By medical waste I mean any kind of waste containing infectious materials generated during treatment (such as soiled dressings, bandages, diagnostic samples, etc.)* | Never has medical waste 0  **BURN INCINERATOR**  2-chanber industrial (800-1000+°C) 1  1-chanber drum/brick 2  **OPEN BURNING**  Flat ground – no protection 3  Pit or protected ground 4  **DUMP WITHOUT BURNING**  Flat ground – no protection 5  Covered pit or pit latrine 6  Open pit – no protection 7  Protected ground or pit 8  **REMOVE OFFSITE**  Stored in a covered container 9  Stored in other protected environment 10  Stored unprotected 12  Same as for sharps items 95  Other (specify) 99 |  |
|  | *INTERVIEWER ONLY:*  *Did the respondent indicate a burn incinerator was used for either medical or sharps waste?*  *Q734=1 or 2*  *Q735=1 or 2* | No 0  Yes 1 | If NO SKIP to Q737 |
| **736a** | Is the incinerator functional today? | No 0  Yes 1  DK 98 | If NO / DK, SKIP To Q737 |
| **736b** | Is there fuel for the incinerator available today? | No 0  Yes 1  DK 98 |  |
| **SUPERVISION** | | | |
| **737** | Does this facility receive any supervision from a higher-level office (such as district, provincial, or central office)? | No 0  Yes 1 | If NO, SKIP to Q801 |
| **738** | When was the last time this facility received a supervision visit from the higher level (district health office or other)? | This month 1  In the last 3 months 2  More than 3 months ago 3  DK 98 | IF >3 MONTHS OR DK, SKIP TO Q801 |
| During the supervision visit, did the supervisor assess the following? | | | |
| **739a** | Pharmacy (e.g., drug stock out, expiry, records, etc.) | No 0  Yes 1 |  |
| **739b** | Staffing (e.g., staff available) | No 0  Yes 1 |  |
| **739c** | Data (e.g., completeness, quality, and timely reporting) | No 0  Yes 1 |  |
| **739d** | Discuss health workers’ clinical skills | No 0  Yes 1 |  |
| **739e** | Help the facility make any decisions based on available health services data | No 0  Yes 1 |  |

| **SECTION 8: BASIC SERVICES, EQUIPMENT & MEDICINES** | | | |
| --- | --- | --- | --- |
| **801** | Do providers in this facility ask clients if they have experienced any intimate-partner violence or sexual violence when they are receiving other services? | No 0  Yes 1 |  |
| **802** | Does this facility have a system for referrals or linkages to other services for victims of gender-based violence (such as to the Lao Women’s Union) if needed? | No 0  Yes 1 |  |
| **803** | Does this facility have a policy to conduct clinical enquiry about intimate partner violence or sexual violence routinely among all patients seeking certain services such as antenatal care and family planning? | No 0  Yes 1 |  |
| Have you or any provider(s) received the following training in the past two years: | | | |
| **804a** | How to ask about intimate partner violence or sexual violence, if patient presents with common signs and symptoms for such violence | No 0  Yes 1 |  |
| **804b** | How to conduct routine enquiry about gender-based violence pr GBV | No 0  Yes 1 |  |
| **BASIC EQUIPMENT** | | | |
| Please tell me if the following basic equipment and supplies used in the provision of client services are available and functional in this facility today.  **IF AVAILABLE, ASK TO SEE IT AND INDICATE IF IT IS FUNCTIONING OR NOT.** | | | |
| **805a** | Is there a sphygmomanometer (blood pressure apparatus) - may be digital or manual sphygmomanometer with stethoscope - in the facility today?  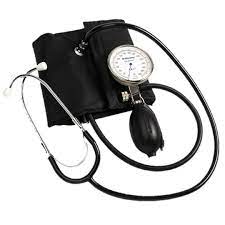  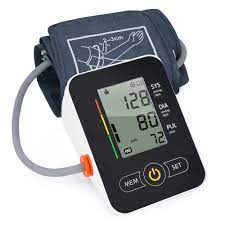 | Not required 0  REQUIRED  Required but not available 1  Yes, observed 2  Yes, reported but not seen 3 | If NO/NA, SKIP to Q806a |
| **805b** | Is the sphygmomanometer (blood pressure apparatus) – digital or manual with a stethoscope - functional today? | No 0  Yes 1  DK 98 |  |
| **806a** | Is there an adult weighing scale in the facility today?  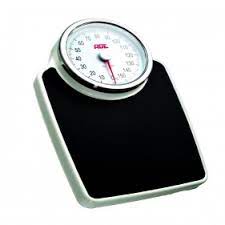 | Not required 0  REQUIRED  Required but not available 1  Yes, observed 2  Yes, reported but not seen 3 | If NO/NA, SKIP to Q807a |
| **806b** | Is the adult weighing scale functional today? | No 0  Yes 1  DK 98 |  |
| **807a** | Is there an infant weighing scale in the facility today?  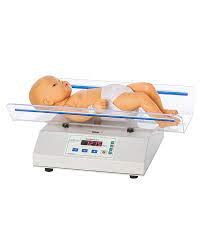 | Not required 0  REQUIRED  Required but not available 1  Yes, observed 2  Yes, reported but not seen 3 | If NO/NA, SKIP to Q808a |
| **807b** | Is the infant weighing scale functional today? | No 0  Yes 1  DK 98 |  |
| **808a** | Is there a stethoscope in the facility today?  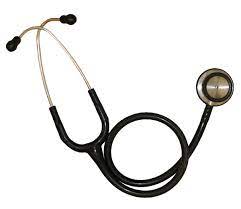 | Not required 0  REQUIRED  Required but not available 1  Yes, observed 2  Yes, reported but not seen 3 | If NO/NA, SKIP to Q809a |
| **808b** | Is the stethoscope functional today? | No 0  Yes 1  DK 98 |  |
| **809a** | Is there a light source (flashlight acceptable) in the facility today?  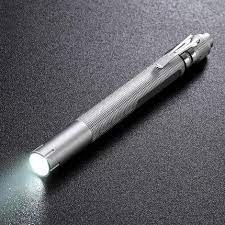 | Not required 0  REQUIRED  Required but not available 1  Yes, observed 2  Yes, reported but not seen 3 | If NO/NA, SKIP to Q810a |
| **809b** | Is the light source (flashlight acceptable) functional today? | No 0  Yes 1  DK 98 |  |
| **810a** | Is there a thermometer (any type of thermometer) in the facility today?  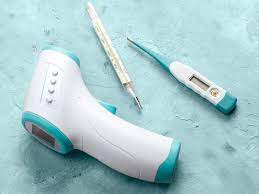 | Not required 0  REQUIRED  Required but not available 1  Yes, observed 2  Yes, reported but not seen 3 | If NO/NA, SKIP to Q811a |
| **810b** | Is the thermometer functional today? | No 0  Yes 1  DK 98 |  |
| **811a** | Are there oxygen concentrators in the facility today?  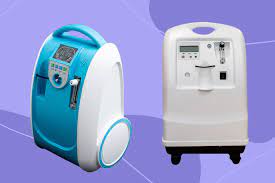 | Not required 0  REQUIRED  Required but not available 1  Yes, observed 2  Yes, reported but not seen 3 | If NO/NA, SKIP to Q812a |
| **811b** | Are the oxygen concentrators functional today? | No 0  Yes 1  DK 98 |  |
| **812a** | Are there oxygen cylinders in the facility today?  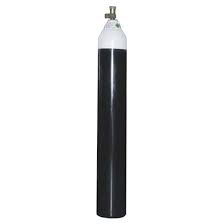 | Not required 0  REQUIRED  Required but not available 1  Yes, observed 2  Yes, reported but not seen 3 | If NO/NA, SKIP to Q813a |
| **812b** | Are the oxygen cylinders functional today? | No 0  Yes 1  DK 98 |  |
| **813a** | Is there a central oxygen supply in the facility today?  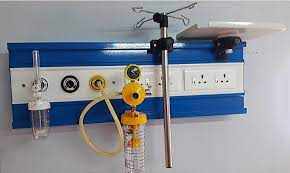 | Not required 0  REQUIRED  Required but not available 1  Yes, observed 2  Yes, reported but not seen 3 | If NO/NA, SKIP to Q814a |
| **813b** | Is the central oxygen supply functional today? | No 0  Yes 1  DK 98 |  |
| **814a** | Is there a flowmeter for oxygen therapy (with humidification)  in the facility today?  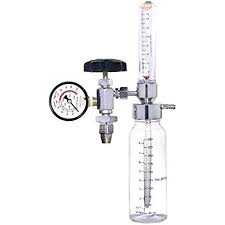 | Not required 0  REQUIRED  Required but not available 1  Yes, observed 2  Yes, reported but not seen 3 | If NO/NA, SKIP to Q815a |
| **814b** | Is the flowmeter for oxygen therapy (with humidification) functional today? | No 0  Yes 1  DK 98 |  |
| **815a** | Is there an oxygen delivery apparatus (key connecting tubes and mask/nasal prongs) in the facility today?  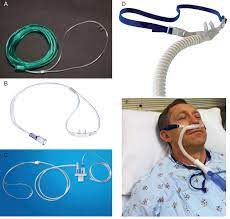 | Not required 0  REQUIRED  Required but not available 1  Yes, observed 2  Yes, reported but not seen 3 | If NO/NA, SKIP to Q816 |
| **815b** | Is the oxygen delivery apparatus (key connecting tubes and mask/nasal prongs) functional today? | No 0  Yes 1  DK 98 |  |
| **816** | At any time during the past 3 months has oxygen been unavailable for any reason? | No 0  Yes 1  No oxygen kept at facility 95  DK 98 |  |
| **817** | Is there a “safety box” available in the facility today?  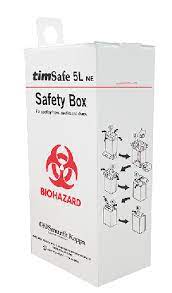  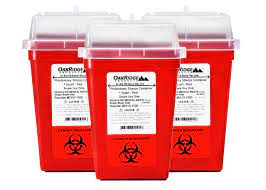 | Not required 0  REQUIRED  Required but not available 1  Yes, observed 2  Yes, reported but not seen 3 |  |
| **818** | Are there information, education, and communication (IEC) materials such as flipcharts for health education available in the facility today (any topic)? | Not required 0  REQUIRED  Required but not available 1  Yes, observed 2  Yes, reported but not seen 3 |  |
| **819** | Mother and child monitoring books (pink book)? | Not required 0  REQUIRED  Required but not available 1  Yes, observed 2  Yes, reported but not seen 3 | If NO/NA, SKIP to Q821a |
| **820** | Does the facility have enough of the mother and child monitoring books (pink book) to distribute to clients? | Yes, in sufficient quantities to distributed to all clients that need them 1  Yes, but not a sufficient amount 2 |  |
| **BASIC MEDICINES** | | | |
| I would like to know if the following medicines are available today in this facility. I would also like to observe the medicines that are available. If any of the medicines I mention is stored in another location in the facility, please tell me where in the facility it is stored so I can go there and verify. | | | |
| **821a** | Normal saline IV solution | Not available today 0  At least one valid 1  Available but not valid 2  Reported available but not seen 3  Not required to stock that medicine / commodity 95 |  |
| **821b** | Ringer’s lactate IV solution | Not available today 0  At least one valid 1  Available but not valid 2  Reported available but not seen 3  Not required to stock that medicine / commodity 95 |  |
| **821c** | 5% dextrose IV solution | Not available today 0  At least one valid 1  Available but not valid 2  Reported available but not seen 3  Not required to stock that medicine / commodity 95 |  |
| **821d** | IV treatment for fungal infections | Not available today 0  At least one valid 1  Available but not valid 2  Reported available but not seen 3  Not required to stock that medicine / commodity 95 |  |
| **821e** | Skin disinfectant | Not available today 0  At least one valid 1  Available but not valid 2  Reported available but not seen 3  Not required to stock that medicine / commodity 95 |  |
| **821f** | Gowns | Not available today 0  At least one valid 1  Available but not valid 2  Reported available but not seen 3  Not required to stock that medicine / commodity 95 |  |
| **821g** | Eye protection (googles, face shields) | Not available today 0  At least one valid 1  Available but not valid 2  Reported available but not seen 3  Not required to stock that medicine / commodity 95 |  |
| **821h** | Medical (surgical and procedural) masks | Not available today 0  At least one valid 1  Available but not valid 2  Reported available but not seen 3  Not required to stock that medicine / commodity 95 |  |
| **821i** | Absorbable suture material  *Used for lacerations or cuts and do not need to be removed.* | Not available today 0  At least one valid 1  Available but not valid 2  Reported available but not seen 3  Not required to stock that medicine / commodity 95 |  |
| **821j** | Ketamine (injection)  *Used as an anesthetic to induce loss of consciousness.* | Not available today 0  At least one valid 1  Available but not valid 2  Reported available but not seen 3  Not required to stock that medicine / commodity 95 |  |
| **821k** | Lidocaine 1% or 2% (anesthesia)  *Used to provide numbness or loss of feeling before medical procedures.* | Not available today 0  At least one valid 1  Available but not valid 2  Reported available but not seen 3  Not required to stock that medicine / commodity 95 |  |
| **821l** | Diazepam (injection)  *Used to treat anxiety, muscle spasms, seizures, or provide sedation before medical procedures*. | Not available today 0  At least one valid 1  Available but not valid 2  Reported available but not seen 3  Not required to stock that medicine / commodity 95 |  |
| **821m** | Glibenclamide tablet | Not available today 0  At least one valid 1  Available but not valid 2  Reported available but not seen 3  Not required to stock that medicine / commodity 95 |  |
| **821n** | Enalapril tablet or alternative ACE inhibitor | Not available today 0  At least one valid 1  Available but not valid 2  Reported available but not seen 3  Not required to stock that medicine / commodity 95 |  |
| **821o** | Insulin regular injection | Not available today 0  At least one valid 1  Available but not valid 2  Reported available but not seen 3  Not required to stock that medicine / commodity 95 |  |
| **821p** | Magnesium sulfate injectable | Not available today 0  At least one valid 1  Available but not valid 2  Reported available but not seen 3  Not required to stock that medicine / commodity 95 |  |
| **821q** | Metformin tablet | Not available today 0  At least one valid 1  Available but not valid 2  Reported available but not seen 3  Not required to stock that medicine / commodity 95 |  |
| **821r** | Hydrochlorothiazide | Not available today 0  At least one valid 1  Available but not valid 2  Reported available but not seen 3  Not required to stock that medicine / commodity 95 |  |
| **821s** | Carbamazepine tablet | Not available today 0  At least one valid 1  Available but not valid 2  Reported available but not seen 3  Not required to stock that medicine / commodity 95 |  |
| **821t** | Simvastatin tablet or other statin | Not available today 0  At least one valid 1  Available but not valid 2  Reported available but not seen 3  Not required to stock that medicine / commodity 95 |  |

| **SECTION 10: ANTENATAL CARE SERVICES** | | | |
| --- | --- | --- | --- |
| **1001** | Does this facility offer antenatal care (ANC) services, either at the facility or through outreach activities? | No 0  Yes 1 | If NO, SKIP to Q1101 |
| IF YES, ASK TO BE SHOWN THE MAIN LOCATION WHERE MATERNAL AND NEWBORN HEALTH SERVICES ARE PROVIDED IN THE FACILITY. FIND THE PERSON MOST KNOWLEDGEABLE ABOUT MATERNAL AND NEWBORN HEALTH SERVICES IN THE FACILITY. INTRODUCE YOURSELF, EXPLAIN THE PURPOSE OF THE SURVEY AND ASK THE FOLLOWING QUESTIONS. | | | |
| As part of ANC services, please tell me if providers in this facility provide the following counseling services  to ANC clients either at the facility or through outreach services (if DK use “NO”): | | | |
| **1002a** | Counseling on recommended minimum of 4 or more ANC visits for each pregnancy | No 0  Yes 1 |  |
| **1002b** | Counseling about healthy eating and physical activity during pregnancy | No 0  Yes 1 |  |
| **1002c** | Counseling on birth preparedness/complication readiness or preparations for delivery | No 0  Yes 1 |  |
| **1002d** | Counseling about postpartum family planning | No 0  Yes 1 |  |
| **1002e** | Counseling about sexually transmitted infections (STIs), including syphilis and HIV/AIDS | No 0  Yes 1 |  |
| **1002f** | Counseling about use of insecticide treated nets (ITNs) to prevent mosquito bites and malaria | No 0  Yes 1 |  |
| **1002g** | Counseling about breastfeeding | No 0  Yes 1 |  |
| **1002h** | Counseling about newborn care | No 0  Yes 1 |  |
| **1002i** | Counseling on postnatal care visits | No 0  Yes 1 |  |
| **1003** | Do providers at this facility provide any outreach health education about pregnancy care in communities? | No 0  Yes 1 | If NO, SKIP to Q1004a |
| Please tell me if the outreach health education about pregnancy care in the communities includes any of the following topics: | | | |
| **1003a** | Individual health education to women and their husbands | No 0  Yes 1  DK 98 |  |
| **1003b** | Detecting antenatal danger signs and referrals to health facilities | No 0  Yes 1  DK 98 |  |
| **1003c** | Birth preparedness and complication readiness (i.e., a plan for emergency transportation, preparing medical documents, payments, etc.) | No 0  Yes 1  DK 98 |  |
| Do ANC providers (at the facility or through outreach services) provide any of the following core services and testing to pregnant women as part of routine ANC services? | | | |
| **1004a** | Calcium supplementation? | No 0  Yes 1 |  |
| **1004b** | Screening for anemia? | No 0  Yes 1 |  |
| **1004c** | Risk factor screening for gestational diabetes mellitus? | No 0  Yes 1 |  |
| **1004d** | Ultrasound scans? | No 0  Yes 1 |  |
| **1004e** | Deworming treatment? | No 0  Yes 1 |  |
| **1004f** | Iron supplementation? | No 0  Yes 1 |  |
| **1004g** | Folic acid supplementation? | No 0  Yes 1 |  |
| **1004h** | Tetanus-toxoid and diphtheria (Td) immunization? | No 0  Yes 1 |  |
| **1004i** | Monitoring for hypertensive disorder of pregnancy? | No 0  Yes 1 |  |
| **1004j** | Intermittent preventive treatment in pregnancy (IPTp) for malaria? | No 0  Yes 1 |  |
| **1004k** | Low dose aspirin for high-risk women to prevent eclampsia? | No 0  Yes 1 |  |
| **1004l** | Antihypertensive drugs to treat high blood pressure? | No 0  Yes 1 |  |
| **1004m** | Testing for any sexually transmitted infections (STIs)? | No 0  Yes 1 |  |
| Do ANC providers in this facility provide any of the following tests from this site to pregnant women as part of ANC?  **IF YES, ASK TO SEE THE TEST KIT OR EQUIPMENT. IF TEST NOT DONE IN ANC, PROBE TO STERMINE IF THE TEST IS DONE ELSEWHERE IN THE FACILITY. CHECK TO SEE IF AT LEAST ONE TEST KIT OF EACH TEST IS VALID AND UNEXPIRED.** | | | |
| **1005a** | HIV rapid diagnostic test | Not required of this facility 0  REQUIRED  Required but never available 1  None available today 2  Reported available, not seen 3  Available, none valid 4  At least one valid 5 |  |
| **1005b** | Urine protein test | Not required of this facility 0  REQUIRED  Required but never available 1  None available today 2  Reported available, not seen 3  Available, none valid 4  At least one valid 5 |  |
| **1005c** | Rapid glucose test | Not required of this facility 0  REQUIRED  Required but never available 1  None available today 2  Reported available, not seen 3  Available, none valid 4  At least one valid 5 |  |
| **1005d** | Any rapid test for hemoglobin | Not required of this facility 0  REQUIRED  Required but never available 1  None available today 2  Reported available, not seen 3  Available, none valid 4  At least one valid 5 |  |
| **1005e** | Syphilis rapid diagnostic test | Not required of this facility 0  REQUIRED  Required but never available 1  None available today 2  Reported available, not seen 3  Available, none valid 4  At least one valid 5 |  |
| Please tell me if the following documents are available in the facility today. **IF AVAILABLE ASK TO SEE THE DOCUMENT.** | | | |
| **1006a** | *National Antenatal Care (ANC) guidelines*? | No 0  Yes, available and observed 1  Yes, reported available but not observed 2 |  |
| **1006b** | Handout from the Antenatal Care (ANC) training? | No 0  Yes, available and observed 1  Yes, reported available but not observed 2 |  |
| **1006c** | Intermittent Preventive Treatment of Malaria during pregnancy (IPTp) guidelines, job aids, or checklists (including wall charts)?  **ACCEPTABLE IF PART OF ANC GUIDELINES** | No 0  Yes, available and observed 1  Yes, reported available but not observed 2 |  |
| **1006d** | Does this facility have a Daily Registration Book for ANC (MCH Form V2.1), use a notebook, or both? | No register or notebook used 0  Official register only used 1  Notebook only used 2  Both an official register and a notebook are used 3 |  |
| Have you or any provider(s) of ANC services: | | | |
| **1007a** | Received any training on ANC in the last two years? | No 0  Yes 1 |  |
| **1007b** | Received any training on Intermittent Preventive Treatment for Malaria during pregnancy (IPTp) in the last two years? | No 0  Yes 1 |  |
| I would like to know if the following items are available in this services area today. I would like to see them. For equipment and instruments, I would also like to know if they are functioning. | | | |
| **1008a** | Fetal stethoscope/Pinnard  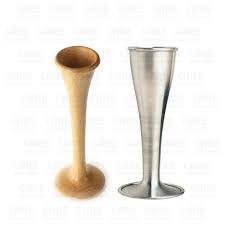 | Not required 0  REQUIRED  Required but not available 1  Yes, observed 2  Yes, reported but not seen 3 |  |
| **1008b** | Fetus stethoscope/traube  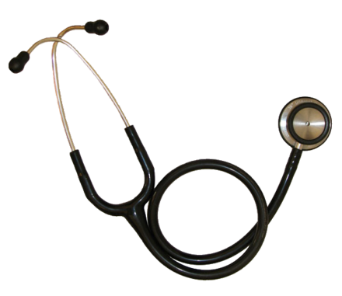 | Not required 0  REQUIRED  Required but not available 1  Yes, observed 2  Yes, reported but not seen 3 |  |
| **1008c** | Ultrasound (2-dimensional ultrasound with transabdominal and transvaginal probes)  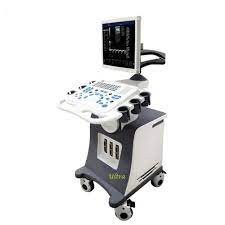 | Not required 0  REQUIRED  Required but not available 1  Yes, observed 2  Yes, reported but not seen 3 |  |
| **1008d** | Fetal monitoring cardiotocography (CTG)  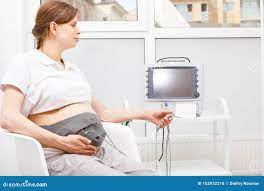 | Not required 0  REQUIRED  Required but not available 1  Yes, observed 2  Yes, reported but not seen 3 |  |
| **1008e** | Fetal Doppler (Sonic Aid) or *Bruits du Coeur foetal (BDC*)  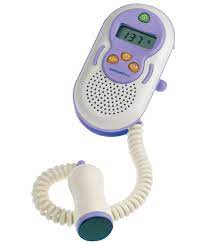 | Not required 0  REQUIRED  Required but not available 1  Yes, observed 2  Yes, reported but not seen 3 |  |
| **1008f** | Pregnancy due date calculator wheel  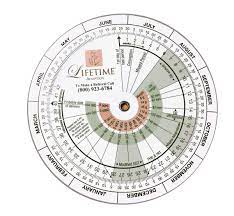 | Not required 0  REQUIRED  Required but not available 1  Yes, observed 2  Yes, reported but not seen 3 |  |
| **1008g** | Measuring tape for uterine fundal height  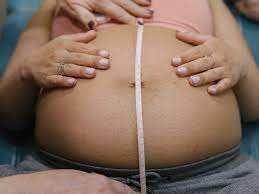 | Not required 0  REQUIRED  Required but not available 1  Yes, observed 2  Yes, reported but not seen 3 |  |
| **1008h** | Urine test strips or AFC  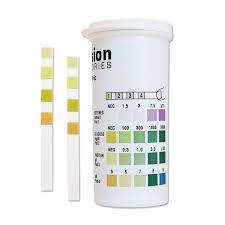 | Not required 0  REQUIRED  Required but not available 1  Yes, observed 2  Yes, reported but not seen 3 |  |
| **1008i** | Height measure  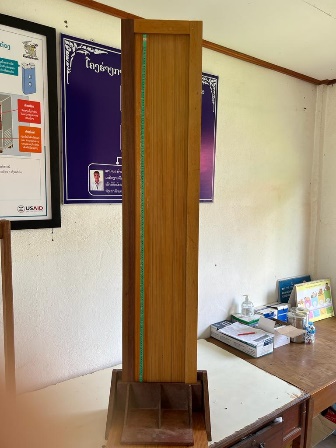 | Not required 0  REQUIRED  Required but not available 1  Yes, observed 2  Yes, reported but not seen 3 |  |
| Please tell me if any of the following medicines are available at this service site or the facility today. I would like to see them.  **CHECK TO SEE IF AT LEAST ONE IS VALID (NOT EXPIRED).** | | | |
| **1009a** | Iron | Not available today 0  At least one valid 1  Available but not valid 2  Reported available but not seen 3  Not required to stock that medicine / commodity 95 |  |
| **1009b** | Folic acid | Not available today 0  At least one valid 1  Available but not valid 2  Reported available but not seen 3  Not required to stock that medicine / commodity 95 |  |
| **1009c** | Td (tetanus-toxoid and diphtheria) vaccine | Not available today 0  At least one valid 1  Available but not valid 2  Reported available but not seen 3  Not required to stock that medicine / commodity 95 |  |
| **1009d** | Deworming (e.g., Mebendazole 500 mg) | Not available today 0  At least one valid 1  Available but not valid 2  Reported available but not seen 3  Not required to stock that medicine / commodity 95 |  |
| **1009e** | Low dose aspirin (81 mg) | Not available today 0  At least one valid 1  Available but not valid 2  Reported available but not seen 3  Not required to stock that medicine / commodity 95 |  |
| **1009f** | Calcium | Not available today 0  At least one valid 1  Available but not valid 2  Reported available but not seen 3  Not required to stock that medicine / commodity 95 |  |
| **1009g** | Hydralazine, nifedipine, or Aldomet (medicines to treat high blood pressure) | Not available today 0  At least one valid 1  Available but not valid 2  Reported available but not seen 3  Not required to stock that medicine / commodity 95 |  |
| **1010** | How many emergency orders for any medicines used in ANC services have you placed in the last three months? | None 0  One 1  Two 2  Three 3  More than 3 4  Not Applicable 97 |  |
| **SECTION 11: OBSTERIC / DELIVERY CARE SERVICES** | | | |
| **1101** | Does this facility offer any delivery care services, including normal delivery, complicated deliveries, basic emergency obstetric care (BEmOC), and/or comprehensive emergency obstetric care (CEmOC)? | No 0  Yes 1 | If NO, SKIP to Q1201 |
| ASK TO BE SHOWN THE MAIN LOCATION WHERE OBSTETRIC AND DELIVERY CARE SERVICES ARE PROVIDED IN THE FACILITY. FIND THE PERSON MOST KNOWLEDGEABLE ABOUT OBSTETRIC AND DELIVERY CARE SERVICES IN THE FACILITY. INTRODUCE YOURSELF, EXPLAIN THE PURPOSE OF THE SURVEY AND ASK THE FOLLOWING QUESTIONS. | | | |
| Please tell me which of the following services are routinely carried out by providers of delivery services in this facility: | | | |
| **1102a** | Administration of corticosteroids (pill or inhaled) to prevent respiratory distress syndrome? | No 0  Yes 1 |  |
| **1102b** | Administration of uterotonic dugs (e.g., oxytocin injection) immediately after birth to all women for post-partum hemorrhage? | No 0  Yes 1 |  |
| **1102c** | Augmentation for prolonged labor?  *By augmentation for prolonged labor I mean the process of stimulation or uterine contraction (both in frequency and duration) that are already present but found to be inadequate.* | No 0  Yes 1 |  |
| **1102d** | Antibiotics for preterm or prolonged PROM (premature rupture of membranes) to prevent infection? | No 0  Yes 1 |  |
| **1102e** | Induction of labor to manage pre-labor rupture of membrane or prolonged pregnancies? | No 0  Yes 1 |  |
| **1102f** | Parenteral administration of antibiotics for mothers? | No 0  Yes 1 |  |
| **1102g** | Administration of parenteral anticonvulsants (e.g., magnesium sulphate) for pre-eclampsia and eclampsia? | No 0  Yes 1 |  |
| **1102h** | Manual removal of placenta | No 0  Yes 1 |  |
| **1102i** | Assisted vaginal delivery (using instruments such as forceps or suction device)? | No 0  Yes 1 |  |
| **1102j** | Removal of retained products of conception | No 0  Yes 1 |  |
| **1102k** | Blood transfusion | No 0  Yes 1 |  |
| **1102l** | Monitoring and management of labor using Partograph? | No 0  Yes 1 |  |
| **1102m** | Detect danger signs for mothers during unplanned home deliveries and refer to health facility? | No 0  Yes 1 |  |
| **1102n** | Does this facility allow birth companions to be present during labor and delivery? | No 0  Yes 1 |  |
| **1102o** | Does this facility provide Caesarean delivery? | No 0  Yes 1 | If NO, SKIP to Q1103a |
| **1102p** | Does this facility have a health professional who can perform caesarean section present in the facility or on call 24 hours a day (including weekends and public holidays)? | No 0  Yes 1 |  |
| **1102q** | Does this facility have an anesthetist (or doctor with anesthetics training) present in the facility or on call 24 hours a day (including weekends and public holidays)? | No 0  Yes 1 |  |
| Are the following documents available in the facility today? | | | |
| **1103a** | The *Pocketbook on Essential Care for Childbirth and Maternal Complications (version June 2020)*? | No 0  Yes, available and observed 1  Yes, reported available but not observed 2 |  |
| **1103b** | CEmOC pocket book? | No 0  Yes, available and observed 1  Yes, reported available but not observed 2 |  |
| **1103c** | Early essential newborn care pocket book? | No 0  Yes, available and observed 1  Yes, reported available but not observed 2 |  |
| **1103d** | Does this facility have an official register (MCH Form V2.1) provided by the MOH or does it use a notebook? | No register or notebook used 0  Official register only used 1  Notebook only used 2  Both an official register and a notebook are used 3 |  |
| I would like to know if the following basic equipment items are available in this services area today. For each equipment or item, please tell me if it is available and functioning. | | | |
| **1104a** | Is the delivery pack available in the facility today?  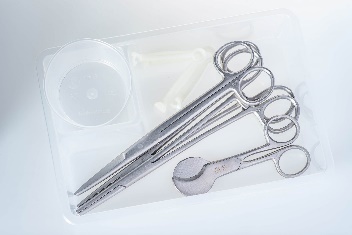  Delivery pack refers to basic delivery kit (e.g., cord clamp, episiotomy scissors, scissors/blade to cut cord, suture material with needle, AND needle holder | Not required 0  REQUIRED  Required but not available 1  Yes, observed 2  Yes, reported but not seen 3 | If NO/NA, SKIP to Q1105a |
| **1104b** | Is a delivery pack functional? | No 0  Yes 1  DK 98 |  |
| **1105a** | Is a cord clamp available in the facility today?  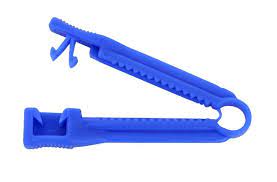 | Not required 0  REQUIRED  Required but not available 1  Yes, observed 2  Yes, reported but not seen 3 | If NO/NA, SKIP to Q1106a |
| **1105b** | Is the cord clamp functional? | No 0  Yes 1  DK 98 |  |
| **1106a** | Are episiotomy scissors available in the facility today?  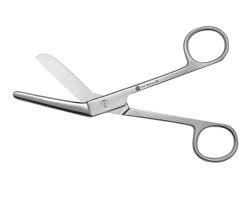 | Not required 0  REQUIRED  Required but not available 1  Yes, observed 2  Yes, reported but not seen 3 | If NO/NA, SKIP to Q1107a |
| **1106b** | Are the episiotomy scissors functional? | No 0  Yes 1  DK 98 |  |
| **1107a** | Are scissors or blade to cut cord available in the facility today?  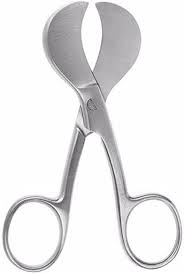 | Not required 0  REQUIRED  Required but not available 1  Yes, observed 2  Yes, reported but not seen 3 | If NO/NA, SKIP to Q1108a |
| **1107b** | Are the scissors or blade to cut cord functional? | No 0  Yes 1  DK 98 |  |
| **1108a** | Is a needle holder available in the facility today?  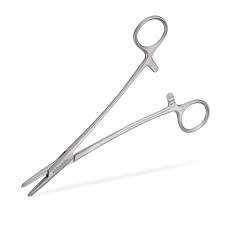 | Not required 0  REQUIRED  Required but not available 1  Yes, observed 2  Yes, reported but not seen 3 | If NO/NA, SKIP to Q1109a |
| **1108b** | Is the needle holder functional? | No 0  Yes 1  DK 98 |  |
| **1109a** | Is an IV catheter N: 20-24 (the set for IV fluid to keeping the vein open) available in the facility today? | Not required 0  REQUIRED  Required but not available 1  Yes, observed 2  Yes, reported but not seen 3 | If NO/NA, SKIP to Q1110a |
| **1109b** | Is the IV catheter N: 20-24 (the set for IV fluid to keeping the vein open) functional today? | No 0  Yes 1  DK 98 |  |
| **1110a** | Is a manual vacuum extractor available in the facility today?  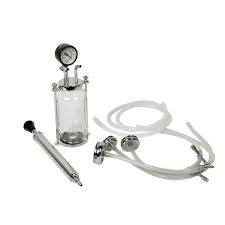 | Not required 0  REQUIRED  Required but not available 1  Yes, observed 2  Yes, reported but not seen 3 | If NO/NA, SKIP to Q1111a |
| **1110b** | Is the manual vacuum extractor functional? | No 0  Yes 1  DK 98 |  |
| **1111a** | Is a vacuum extractor (Kiwi ventouse) available in the facility today?  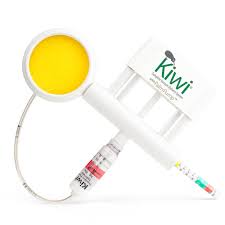 | Not required 0  REQUIRED  Required but not available 1  Yes, observed 2  Yes, reported but not seen 3 | If NO/NA, SKIP to Q1112a |
| **1111b** | Is a vacuum extractor (Kiwi ventouse) functional today? | No 0  Yes 1  DK 98 |  |
| **1112a** | Is a vacuum aspirator or D&C kit available in the facility today?  Vacuum aspirator:  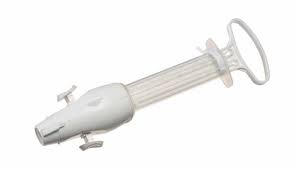  Dilation & Curettage Kit:  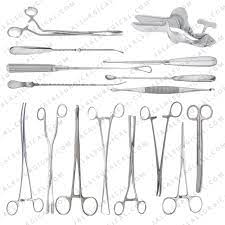 | Not required 0  REQUIRED  Required but not available 1  Yes, observed 2  Yes, reported but not seen 3 | If NO/NA, SKIP to Q1113a |
| **1112b** | Is the vacuum aspirator or D&C kit functional? | No 0  Yes 1  DK 98 |  |
| **1113a** | Is a delivery bed available in the facility today?  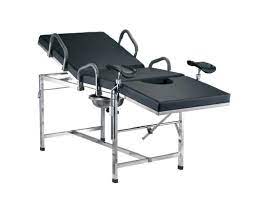 | Not required 0  REQUIRED  Required but not available 1  Yes, observed 2  Yes, reported but not seen 3 | If NO/NA, SKIP to Q1114a |
| **1113b** | Is the delivery bed functional? | No 0  Yes 1  DK 98 |  |
| **1114a** | Is a delivery table or trolley (for setting up delivery sets) available in the facility today?  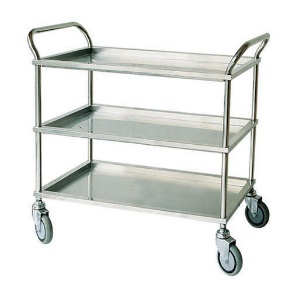 | Not required 0  REQUIRED  Required but not available 1  Yes, observed 2  Yes, reported but not seen 3 | If NO/NA, SKIP to Q1115a |
| **1114b** | Is the delivery table or trolley (for setting up delivery sets) functional today? | No 0  Yes 1  DK 98 |  |
| **1115a** | Is a speculum available in the facility today?  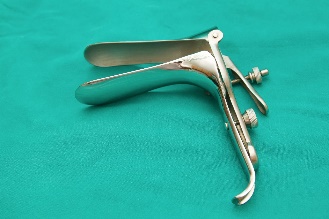 | Not required 0  REQUIRED  Required but not available 1  Yes, observed 2  Yes, reported but not seen 3 | If NO/NA, SKIP to Q1116 |
| **1115b** | Is the speculum functional? | No 0  Yes 1  DK 98 |  |
| **1116** | Is a blank partograph available in the facility today?  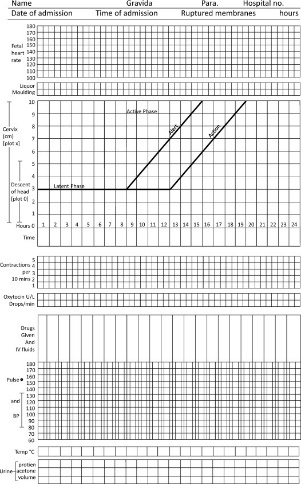 | Yes, available and observed 1  Yes, reported available but not observed 2  No, not available 3 |  |
| **1117** | Are clean dry towels available in the facility today? | Yes, available and observed 1  Yes, reported available but not observed 2  No, not available 3 |  |
| **1118** | Are single use towels available today? | Yes, available and observed 1  Yes, reported available but not observed 2  No, not available 3 |  |
| **1119** | Is hand gel readily available in pre-delivery, delivery, and post-delivery rooms) today? | Yes, available and observed 1  Yes, reported available but not observed 2  No, not available 3 |  |
| **1120** | Is suture material with needle available in the facility today? | Yes, available and observed 1  Yes, reported available but not observed 2  No, not available 3 |  |
| Does this facility stock any of the following medicines and commodities in this service site today?  **CHECK TO SEE IF AT LEAST ONE OF EACH MEDICINE /COMMODITY IS VALID (NOT EXPIRED).** | | | |
| **1121a** | Dexamethasone injection | Not available today 0  At least one valid 1  Available but not valid 2  Reported available but not seen 3  Not required to stock that medicine / commodity 95 |  |
| **1121b** | Magnesium sulphate injectable | Not available today 0  At least one valid 1  Available but not valid 2  Reported available but not seen 3  Not required to stock that medicine / commodity 95 |  |
| **1121c** | Oxytocin injection | Not available today 0  At least one valid 1  Available but not valid 2  Reported available but not seen 3  Not required to stock that medicine / commodity 95 | If AT LEAST ONE VALID, ASK Q1021d  ALL OTHER RESPONSES SKIP TO Q1121e |
| **1121d** | Is oxytocin in cold storage? | No 0  Yes 1 |  |
| **1121e** | Misoprostol 200µg tablets | Not available today 0  At least one valid 1  Available but not valid 2  Reported available but not seen 3  Not required to stock that medicine / commodity 95 |  |
| **1121f** | Aspirin  Ampicillin powder for injection | Not available today 0  At least one valid 1  Available but not valid 2  Reported available but not seen 3  Not required to stock that medicine / commodity 95 |  |
| **1121g** | Gentamicin injection | Not available today 0  At least one valid 1  Available but not valid 2  Reported available but not seen 3  Not required to stock that medicine / commodity 95 |  |
| **1121h** | Aminophylline | Not available today 0  At least one valid 1  Available but not valid 2  Reported available but not seen 3  Not required to stock that medicine / commodity 95 |  |
| **1121i** | Phenobarbital | Not available today 0  At least one valid 1  Available but not valid 2  Reported available but not seen 3  Not required to stock that medicine / commodity 95 |  |
| **1121j** | Metronidazole injection | Not available today 0  At least one valid 1  Available but not valid 2  Reported available but not seen 3  Not required to stock that medicine / commodity 95 |  |
| **1121k** | Methergine | Not available today 0  At least one valid 1  Available but not valid 2  Reported available but not seen 3  Not required to stock that medicine / commodity 95 |  |
| **1121l** | Misoprostol | Not available today 0  At least one valid 1  Available but not valid 2  Reported available but not seen 3  Not required to stock that medicine / commodity 95 |  |
| **1121m** | Tranexamic acid | Not available today 0  At least one valid 1  Available but not valid 2  Reported available but not seen 3  Not required to stock that medicine / commodity 95 |  |
| **1121n** | Anesthesia | Not available today 0  At least one valid 1  Available but not valid 2  Reported available but not seen 3  Not required to stock that medicine / commodity 95 |  |
| **1121o** | Intravenous solution with infusion set  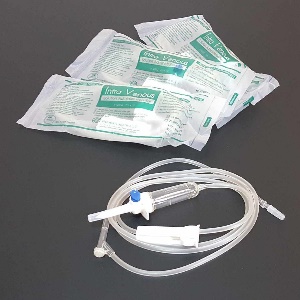 | Not available today 0  At least one valid 1  Available but not valid 2  Reported available but not seen 3  Not required to stock that medicine / commodity 95 |  |
| **1122** | How many emergency orders for any maternal health medicines have you placed in the last three months? | None 0  One 1  Two 2  Three 3  More than 3 4  Not Applicable 97 |  |
| Are any of the following medicines available in this service area or facility today? | | | |
| **1123a** | Thiopental (powder) | Not available today 0  At least one valid 1  Available but not valid 2  Reported available but not seen 3  Not required to stock that medicine / commodity 95 |  |
| **1123b** | Suxamethonium bromide (powder) | Not available today 0  At least one valid 1  Available but not valid 2  Reported available but not seen 3  Not required to stock that medicine / commodity 95 |  |
| **1123c** | Atropine (injection) | Not available today 0  At least one valid 1  Available but not valid 2  Reported available but not seen 3  Not required to stock that medicine / commodity 95 |  |
| **1123d** | Halothane (inhalation) | Not available today 0  At least one valid 1  Available but not valid 2  Reported available but not seen 3  Not required to stock that medicine / commodity 95 |  |
| **1123e** | Bupivacaine (injection) | Not available today 0  At least one valid 1  Available but not valid 2  Reported available but not seen 3  Not required to stock that medicine / commodity 95 |  |
| **1123f** | Lidocaine 5% (heavy spinal solution) | Not available today 0  At least one valid 1  Available but not valid 2  Reported available but not seen 3  Not required to stock that medicine / commodity 95 |  |
| **1123g** | Epinephrine (injection) | Not available today 0  At least one valid 1  Available but not valid 2  Reported available but not seen 3  Not required to stock that medicine / commodity 95 |  |
| **1123h** | Ephedrine (injection) | Not available today 0  At least one valid 1  Available but not valid 2  Reported available but not seen 3  Not required to stock that medicine / commodity 95 |  |
| **1124** | Have you or any provider(s) of child health services received any training in Comprehensive Emergency Obstetric Care (CEmOC) in the last two years? | No 0  Yes 1 |  |
| **1125** | Have you or any provider(s) of child health services received any training in Basic Emergency Obstetric Care (BEmOC) in the last two years? | No 0  Yes 1 |  |
| **SECTION 12: NEWBORN CARE SERVICES** | | | |
| **1201** | Does this facility offer any newborn care services? | No 0  Yes 1 | If NO, SKIP to Q1301 |
| Please tell me which of the following services are routinely carried out by providers of newborn care services in this facility: | | | |
| **1202a** | Neonatal resuscitation with bag and mask? | No 0  Yes 1 |  |
| **1202b** | Administration of injectable antibiotics for neonatal sepsis? | No 0  Yes 1 |  |
| **1202c** | Hygienic cord care (cut with sterile item and apply disinfectant to tip and stump, and no application of other substance)? | No 0  Yes 1 |  |
| **1202d** | Thermal protection (drying baby immediately after birth and wrapping)? | No 0  Yes 1 |  |
| **1202e** | Counseling on immediate and exclusive breastfeeding? | No 0  Yes 1 |  |
| **1202f** | Provided instruction on KMC (Kangaroo mother care) for premature/very small babies? | No 0  Yes 1 |  |
| **1202g** | Detection and referral for low birth-weight babies (<2000g)? | No 0  Yes 1 |  |
| **1202h** | Management of newborns with sepsis, jaundice, severe asphyxia, preterm birth, malformation, seizures? | No 0  Yes 1 |  |
| **1202i** | Administration of magnesium sulphate for fetal neuro-protection in preterm babies? | No 0  Yes 1 |  |
| Have you or any provider(s) of delivery services: | | | |
| **1203a** | Received any training in newborn resuscitation using the newborn bag and mask or neonatal resuscitation in the last two years? | No 0  Yes 1 |  |
| **1203b** | Apart from newborn resuscitation, received training in essential childbirth care in the last two years. | No 0  Yes 1 |  |
| I would like to know if the following basic equipment items are available in this services area today. For each equipment or item, please tell me if it is available and functioning. | | | |
| **1204a** | Is a suction catheter (for suction apparatus) for suctioning newborn available in the facility today? | Not required 0  REQUIRED  Required but not available 1  Yes, observed 2  Yes, reported but not seen 3 | If NO/NA, SKIP to Q1205a |
| **1204b** | Is the suction catheter (for suction apparatus) for suctioning newborn functional? | No 0  Yes 1  DK 98 |  |
| **1205a** | Is a suction bulb (single use) available in the facility today? | Not required 0  REQUIRED  Required but not available 1  Yes, observed 2  Yes, reported but not seen 3 | If NO/NA, SKIP to Q1206a |
| **1205b** | Is the suction bulb (single use) functional? | No 0  Yes 1  DK 98 |  |
| **1206a** | Is a suction bulb (sterilizable multiple use) available in the facility today? | Not required 0  REQUIRED  Required but not available 1  Yes, observed 2  Yes, reported but not seen 3 | If NO/NA, SKIP to Q1207a |
| **1206b** | Is the suction bulb (sterilizable multiple use) functional? | No 0  Yes 1  DK 98 |  |
| **1207a** | Is a continuous positive airway pressure therapy (CPAP) machine available in the facility today?  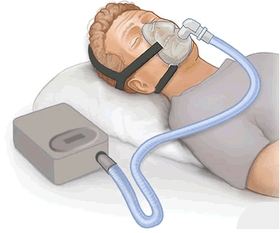 | Not required 0  REQUIRED  Required but not available 1  Yes, observed 2  Yes, reported but not seen 3 | If NO/NA, SKIP to Q1208a |
| **1207b** | Is the continuous positive airway pressure therapy (CPAP) machine functional today? | No 0  Yes 1  DK 98 |  |
| **1208a** | Is a nasogastric tube (for child/newborn) available in the facility today?  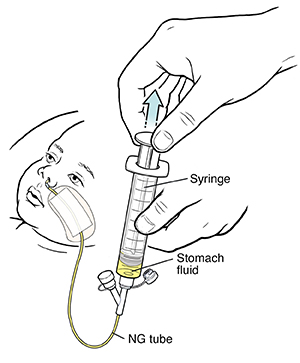 | Not required 0  REQUIRED  Required but not available 1  Yes, observed 2  Yes, reported but not seen 3 | If NO/NA, SKIP to Q1209a |
| **1208b** | Is a nasogastric tube (for child/newborn) functional today? | No 0  Yes 1  DK 98 |  |
| **1209a** | Is an incubator available in the facility today? | Not required 0  REQUIRED  Required but not available 1  Yes, observed 2  Yes, reported but not seen 3 | If NO/NA, SKIP to Q1210a |
| **1209b** | Is the incubator functional? | No 0  Yes 1  DK 98 |  |
| **1210a** | Is a resuscitation table (with heat source) for newborn resuscitation available in the facility today? | Not required 0  REQUIRED  Required but not available 1  Yes, observed 2  Yes, reported but not seen 3 | If NO/NA, SKIP to Q1211a |
| **1210b** | Is the resuscitation table (with heat source) (for newborn resuscitation functional? | No 0  Yes 1  DK 98 |  |
| **1211a** | Is a newborn bag and mask size 1 for term babies (for newborn resuscitation) available in the facility today? | Not required 0  REQUIRED  Required but not available 1  Yes, observed 2  Yes, reported but not seen 3 | If NO/NA, SKIP to Q1212a |
| **1211b** | Is the newborn bag and mask size 1 for term babies (for newborn resuscitation) functional? | No 0  Yes 1  DK 98 |  |
| **1212a** | Is a newborn bag and mask size 0 for pre-term babies (for newborn resuscitation) available in the facility today? | Not required 0  REQUIRED  Required but not available 1  Yes, observed 2  Yes, reported but not seen 3 | If NO/NA, SKIP to Q1213a |
| **1212b** | Is the newborn bag and mask size 0 for pre-term babies (for newborn resuscitation) functional? | No 0  Yes 1  DK 98 |  |
| **1213a** | Is an electric suction pump (for suction apparatus) available in the facility today?  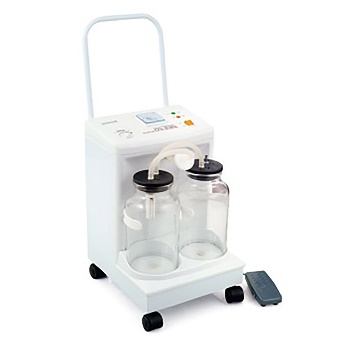 | Not required 0  REQUIRED  Required but not available 1  Yes, observed 2  Yes, reported but not seen 3 | If NO/NA, SKIP to Q1214a |
| **1213b** | Is the electric suction pump (for suction apparatus) functional? | No 0  Yes 1  DK 98 |  |
| **1214a** | Is a stethoscope (baby/infant/newborn) available in the facility today? | Not required 0  REQUIRED  Required but not available 1  Yes, observed 2  Yes, reported but not seen 3 | If NO/NA, SKIP to Q1215a |
| **1214b** | Is a stethoscope (baby) functional today? | No 0  Yes 1  DK 98 |  |
| **1215a** | Is a measuring tape for newborn height available in the facility today? | Not required 0  REQUIRED  Required but not available 1  Yes, observed 2  Yes, reported but not seen 3 | If NO/NA, SKIP to Q1216a |
| **1215b** | Is a measuring tape for newborn height functional today? | No 0  Yes 1  DK 98 |  |
| Does this facility routinely observe any of the following postpartum or newborn related practices? | | | |
| **1216a** | Placing newborn to the abdomen (skin-to-skin) | No 0  Yes 1  DK 98 |  |
| **1216b** | Immediate and thorough drying and wrapping newborns to keep them warm | No 0  Yes 1  DK 98 |  |
| **1216c** | Initiation of breastfeeding within the first hour | No 0  Yes 1  DK 98 |  |
| **1216d** | Routine, complete (head-to-toe) examination of newborn | No 0  Yes 1  DK 98 |  |
| **1216e** | Suction of the newborn by means of catheter | No 0  Yes 1  DK 98 |  |
| **1216f** | Suction of the newborn by means of suction bulb or penguin sucker | No 0  Yes 1  DK 98 |  |
| **1216g** | Weigh the newborn immediately | No 0  Yes 1  DK 98 |  |
| **1216h** | Administer Vitamin K to the newborn | No 0  Yes 1  DK 98 |  |
| **1216i** | Apply Tetracycline eye ointment to both eyes | No 0  Yes 1  DK 98 |  |
| **1216j** | Give the newborn prelacteal liquids (first milk or colostrum) | No 0  Yes 1  DK 98 |  |
| **1216k** | Give the newborn oral polio vaccine (OPV)/polio zero vaccine prior to discharge | No 0  Yes 1  DK 98 |  |
| **1216l** | Give the newborn BCG after birth (and prior to discharge) | No 0  Yes 1  DK 98 |  |
| **1216m** | Give the newborn HepB after birth (and prior to discharge) | No 0  Yes 1  DK 98 |  |
| **1216n** | Provide counseling on post-partum family planning prior to discharge | No 0  Yes 1  DK 98 |  |
| Does this facility stock any of the following medicines and commodities in this service site today?  **CHECK TO SEE IF AT LEAST ONE OF EACH MEDICINE /COMMODITY IS VALID (NOT EXPIRED).** | | | |
| **1217a** | Antibiotic eye ointment for newborn | Not available today 0  At least one valid 1  Available but not valid 2  Reported available but not seen 3  Not required to stock that medicine / commodity 95 |  |
| **1217b** | Skin disinfectant (alcohol, 90/75 disinfectant) | Not available today 0  At least one valid 1  Available but not valid 2  Reported available but not seen 3  Not required to stock that medicine / commodity 95 |  |
| **1217c** | Vitamin K | Not available today 0  At least one valid 1  Available but not valid 2  Reported available but not seen 3  Not required to stock that medicine / commodity 95 |  |
| **1217d** | Hepatitis B vaccine | Not available today 0  At least one valid 1  Available but not valid 2  Reported available but not seen 3  Not required to stock that medicine / commodity 95 |  |
| **1217e** | BCG vaccine | Not available today 0  At least one valid 1  Available but not valid 2  Reported available but not seen 3  Not required to stock that medicine / commodity 95 |  |
| **1217f** | Ampicillin – injectable | Not available today 0  At least one valid 1  Available but not valid 2  Reported available but not seen 3  Not required to stock that medicine / commodity 95 |  |
| **1217g** | Cloxacillin injectable | Not available today 0  At least one valid 1  Available but not valid 2  Reported available but not seen 3  Not required to stock that medicine / commodity 95 |  |
| **1217h** | Gentamicin – injectable | Not available today 0  At least one valid 1  Available but not valid 2  Reported available but not seen 3  Not required to stock that medicine / commodity 95 |  |
| **1217i** | Aminophylline | Not available today 0  At least one valid 1  Available but not valid 2  Reported available but not seen 3  Not required to stock that medicine / commodity 95 |  |
| **1217j** | Phenobarbital | Not available today 0  At least one valid 1  Available but not valid 2  Reported available but not seen 3  Not required to stock that medicine / commodity 95 |  |
| **1218** | How many emergency orders for any newborn care medicines have you placed in the last three months? | None 0  One 1  Two 2  Three 3  More than 3 4  Not Applicable 97 |  |
| **SECTION 13: POSTNATAL CARE** | | | |
| **1301** | Does this facility provide post-partum and post-natal care for mothers and newborns?  (Refers to care provided more than 24 hours after a facility delivery OR within 24 hours after a home delivery) | No 0  Yes 1 | If NO, SKIP to Q1401 |
| Please tell me if this facility provides the following services for mothers in the first 24 hours starting from the first hour after birth: | | | |
| **1302a** | Blood pressure measurement | No 0  Yes 1 |  |
| **1302b** | Assessment of vaginal bleeding | No 0  Yes 1 |  |
| **1302c** | Assessment of uterine contractions | No 0  Yes 1 |  |
| **1302d** | Assessment of urine void | No 0  Yes 1 |  |
| **1302e** | Assessment of fundal height | No 0  Yes 1 |  |
| **1302f** | Assessment of temperature | No 0  Yes 1 |  |
| **1302g** | Assessment of heart rate (pulse) | No 0  Yes 1 |  |
| Please tell me if this facility provides the following counseling for mothers in the first 24 hours starting from the first hour after birth: | | | |
| **1303a** | Physiological recovery | No 0  Yes 1 |  |
| **1303b** | Danger signs to look for | No 0  Yes 1 |  |
| **1303c** | Maternal nutrition | No 0  Yes 1 |  |
| **1303d** | Maternal hygiene | No 0  Yes 1 |  |
| **1303e** | Malaria protection | No 0  Yes 1 |  |
| **1303f** | Family planning | No 0  Yes 1 |  |
| **1303g** | Breastfeeding techniques | No 0  Yes 1 |  |
| Please tell me if this facility provides the following services for newborns in the first 24 hours starting from the first hour after birth: | | | |
| **1304a** | Assessment of newborn feeding | No 0  Yes 1 |  |
| **1304b** | Assessment of history of convulsions | No 0  Yes 1 |  |
| **1304c** | Assessment of fast breathing | No 0  Yes 1 |  |
| **1304d** | Assessment of chest-in drawing | No 0  Yes 1 |  |
| **1304e** | Assessment of spontaneous movement | No 0  Yes 1 |  |
| **1304f** | Assessment of fever | No 0  Yes 1 |  |
| **1304g** | Assessment of low body temperature | No 0  Yes 1 |  |
| **1304h** | Assessment of jaundice <24 hours | No 0  Yes 1 |  |
| **1304i** | Assessment of yellow palms and soles at any time | No 0  Yes 1 |  |
| **1304j** | Assessment of dry cord care | No 0  Yes 1 |  |
| **1304k** | Assessment of signs of cord infection | No 0  Yes 1 |  |
| **1305** | Does the facility provide iron and folic acid supplementation and Vitamin B1 for postpartum lactating women? | No 0  Yes 1 |  |
| **SECTION 16: DIAGNOSTICS/LABORATORY SERVICES** | | | |
| **1601** | Does this facility conduct any diagnostic testing including any rapid diagnostic testing? | No 0  Yes 1 | If NO, SKIP to Q1701 |
| Ask to be shown the main laboratory or location in the facility where most testing is done to start data collection. Find the person most knowledgeable about the diagnostic or laboratory services in the facility. Introduce yourself, explain the purpose of the survey and ask the following questions. | | | |
| Does this facility offer any of the following tests on-site: | | | |
| **1602a** | Rapid syphilis testing | No 0  Yes 1  Not required to provide this test at this type of facility 96 |  |
| **1602b** | HIV rapid testing | No 0  Yes 1  Not required to provide this test at this type of facility 96 |  |
| **1602c** | Urine rapid tests for pregnancy | No 0  Yes 1  Not required to provide this test at this type of facility 96 |  |
| **1602d** | Urine protein dipstick testing | No 0  Yes 1  Not required to provide this test at this type of facility 96 |  |
| **1602e** | Urine glucose dipstick testing | No 0  Yes 1  Not required to provide this test at this type of facility 96 |  |
| **1602f** | Urine ketone dipstick testing | No 0  Yes 1  Not required to provide this test at this type of facility 96 |  |
| I would like to know if the following items for rapid diagnostic testing are available or not available today.  CHECK TO SEE IF AT LEAST ONE OF EACH RDT IS VALID (NOT EXPIRED). | | | |
| **1603a** | Malaria rapid diagnostic kit | Not available today 0  At least one valid 1  Available but not valid 2  Reported available but not seen 3  Not required to stock that medicine / commodity 95 |  |
| **1603b** | Syphilis rapid test kit | Not available today 0  At least one valid 1  Available but not valid 2  Reported available but not seen 3  Not required to stock that medicine / commodity 95 |  |
| **1603c** | HIV rapid test kit | Not available today 0  At least one valid 1  Available but not valid 2  Reported available but not seen 3  Not required to stock that medicine / commodity 95 |  |
| **1603d** | Urine pregnancy test kit | Not available today 0  At least one valid 1  Available but not valid 2  Reported available but not seen 3  Not required to stock that medicine / commodity 95 |  |
| **1603e** | Dipsticks for urine protein | Not available today 0  At least one valid 1  Available but not valid 2  Reported available but not seen 3  Not required to stock that medicine / commodity 95 |  |
| **1603f** | Dipsticks for urine glucose | Not available today 0  At least one valid 1  Available but not valid 2  Reported available but not seen 3  Not required to stock that medicine / commodity 95 |  |
| **1603g** | Dipsticks for urine ketone bodies | Not available today 0  At least one valid 1  Available but not valid 2  Reported available but not seen 3  Not required to stock that medicine / commodity 95 |  |
| **1603h** | Filet paper for collecting DBS  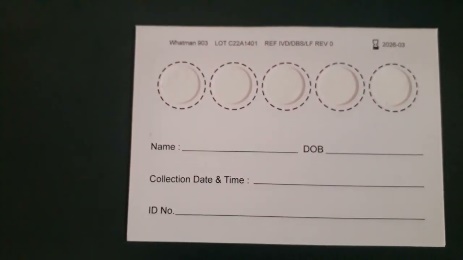 | Not available today 0  At least one valid 1  Available but not valid 2  Reported available but not seen 3  Not required to stock that medicine / commodity 95 |  |
| Does this facility offer the following tests onsite or offsite: | | | |
| **1604a** | Blood glucose tests using a glucometer  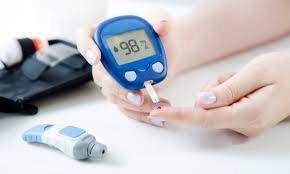 | Not required to offer 0  REQUIRED  Required but not offered 1  Yes, onsite 2  Yes, offsite 3 |  |
| **1604b** | Hemoglobin testing | Not required to offer 0  REQUIRED  Required but not offered 1  Yes, onsite 2  Yes, offsite 3 |  |
| **1604c** | General microscopy/wet mounts | Not required to offer 0  REQUIRED  Required but not offered 1  Yes, onsite 2  Yes, offsite 3 |  |
| **1604d** | Malaria smear tests | Not required to offer 0  REQUIRED  Required but not offered 1  Yes, onsite 2  Yes, offsite 3 |  |
| I would like to know if the following general equipment items are available and functional today. ASK TO SEE THE ITEMS. | | | |
| **1605a** | Light microscope available? | Not required 0  REQUIRED  Required but not available 1  Yes, observed 2  Yes, reported but not seen 3 | If NO, SKIP to Q1606a |
| **1605b** | Light microscope functional? | No 0  Yes 1  DK 98 |  |
| **1606a** | Glass slides and cover slips available? | Not required 0  REQUIRED  Required but not available 1  Yes, observed 2  Yes, reported but not seen 3 | If NO, SKIP to Q1607a |
| **1606b** | Glass slides and cover slips functional? | No 0  Yes 1  DK 98 |  |
| **1607a** | Refrigerator available? | Not required 0  REQUIRED  Required but not available 1  Yes, observed 2  Yes, reported but not seen 3 | If NO, SKIP to Q1608a |
| **1607b** | Refrigerator functional? | No 0  Yes 1  DK 98 |  |
| **1608a** | Glucometer available?  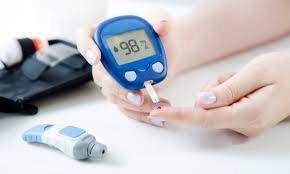 | Not required 0  REQUIRED  Required but not available 1  Yes, observed 2  Yes, reported but not seen 3 | If NO, SKIP to Q1609a |
| **1608b** | Glucometer functional? | No 0  Yes 1  DK 98 |  |
| **1609a** | Glucometer test strips (with valid expiration date) available?  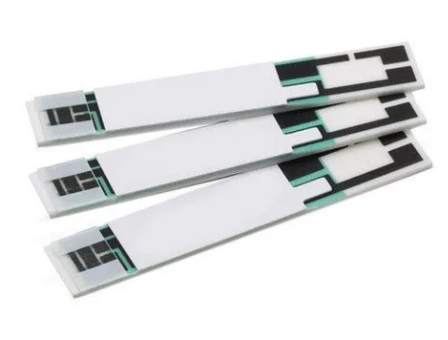 | Not required 0  REQUIRED  Required but not available 1  Yes, observed 2  Yes, reported but not seen 3 | If NO, SKIP to Q1610a |
| **1609b** | Glucometer test strips (with valid expiration date) functional? | No 0  Yes 1  DK 98 |  |
| **1610a** | Colorimeter or hemoglobinometer available? | Not required 0  REQUIRED  Required but not available 1  Yes, observed 2  Yes, reported but not seen 3 | If NO, SKIP to Q1611a |
| **1610b** | Colorimeter or hemoglobinometer functional? | No 0  Yes 1  DK 98 |  |
| **1611a** | HemoCue available?  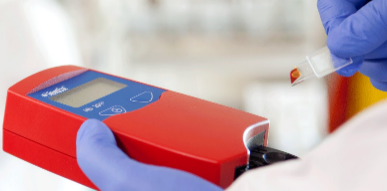 | Not required 0  REQUIRED  Required but not available 1  Yes, observed 2  Yes, reported but not seen 3 | If NO, SKIP to Q1612a |
| **1611b** | HemoCue functional? | No 0  Yes 1  DK 98 |  |
| **1612a** | Incubator available? | Not required 0  REQUIRED  Required but not available 1  Yes, observed 2  Yes, reported but not seen 3 | If NO, SKIP to Q1613 |
| **1612b** | Incubator functional? | No 0  Yes 1  DK 98 |  |
| **1613** | Does thus facility perform diagnostic x-rays, ultrasound, or computerized tomography? | No 0  Yes 1 | If No, SKIP to Q1701 |
| Please tell me if the following imaging equipment items are available and functional today. ASK TO SEE THE ITEMS. | | | |
| **1614a** | X-ray machine available? | Not available 0  Reported, not seen 1  Observed 2  Not required at this facility type 96 | If NO, SKIP to Q1615a |
| **1614b** | X-ray machine functional? | No 0  Yes 1  DK 98 |  |
| **1615a** | Ultrasound equipment available? | Not available 0  Reported, not seen 1  Observed 2  Not required at this facility type 96 | If NO, SKIP to Q1616a |
| **1615b** | Ultrasound equipment functional? | No 0  Yes 1  DK 98 |  |
| **1616a** | Electrocardiogram (ECG) available? | Not available 0  Reported, not seen 1  Observed 2  Not required at this facility type 96 | If NO, SKIP to Q1701 |
| **1616b** | Electrocardiogram (ECG) functional? | No 0  Yes 1  DK 98 |  |
| **SECTION 17: BLOOD TRANSFUSION** | | | |
| ASK TO BE SHOWN THE MAIN LOCATION WHERE BLOOD TRANSFUSION SERVICES ARE PROVIDED IN THE FACILITY. FIND THE PERSON MOST KNOWLEDGEABLE ABOUT CHILD PREVENTIVE AND CARE SERVICES IN THE FACILITY. INTRODUCE YOURSELF, EXPLAIN THE PURPOSE OF THE SURVEY AND ASK THE FOLLOWING QUESTIONS. | | | |
| **1701** | Does this facility offer blood transfusion services? | No 0  Yes 1 | If NO, SKIP to Q1801 |
| **1702** | Have there been any interruptions in blood availability during the past 3 months? | No 0  Yes 1 |  |
| **1703** | Does this facility obtain blood for a national, regional or provincial blood center? | No 0  Yes 1 |  |
| **1704** | Does this facility obtain ANY blood from sources other than the national, regional, or provincial blood center? | No 0  Yes 1 |  |
| **1705** | Does any place in this facility do blood screening for infectious diseases prior to transfusion? | No 0  Yes 1 | If NO, SKIP to Q1707 |
| Please tell me if the blood that is transfused in the facility is “always”, “sometimes”, “rarely”, or “never” screened for any of the following infectious diseases: | | | |
| **1706a** | HIV | Never 0  Rarely 1  Sometimes 2  Always 3  Don’t Know 98 |  |
| **1706b** | Syphilis | Never 0  Rarely 1  Sometimes 2  Always 3  Don’t Know 98 |  |
| **1706c** | Hepatitis B | Never 0  Rarely 1  Sometimes 2  Always 3  Don’t Know 98 |  |
| **1706d** | Hepatitis C | Never 0  Rarely 1  Sometimes 2  Always 3  Don’t Know 98 |  |
| **1707** | Does this facility have a refrigerator available and functioning in this services area for the storage of blood? | No refrigerator available 0  Not functioning 1  Available and functional 2  Available not functional 3  Available don’t know if functioning 4 | IF NO or NOT FUNCTIONING, SKIP to Q1709 |
| **1708a** | Is the temperature of the refrigerator monitored at least once every 24 hours?    **IF YES: PLEASE ASK TO SEE THE LOG USED TO RECORD THE TEMPERATURE** | No 0  Yes, log observed 1  Yes, log reported not seen 2 | If NO, SKIP to Q1709 |
| **1708b** | Has the temperature log been completed for the last 30 days?  **PLEASE REVIEW LOG AND CHECK FOR COMPLETENESS (TEMPERATURE RECORDED AT LEAST ONCE EVERY 24 HOURS DURING THE LAST 30 DAYS)** | No 0  Yes, partially 1  Yes 2 | If NO, SKIP to Q1709 |
| **1708c** | Has the temperature been out of the range 2 to 8°C inclusive in the last 30 days?  **PLEASE CHECK THE TEMPERATURE RECORD AND VERIFY THE TEMPERATURE FOR THE LAST 30 WORKING DAYS IN ORDER TO ANSWER THE QUESTION** | Record not available 0  Out of Range 1  Reported in range but not seen 2  Observed in range 3 | If NA, SKIP to Q1709 |
| **1709** | Do you have any guidelines on the appropriate use of blood and safe transfusion practices?  **IF AVAILABLE, ASK TO SEE THE DOCUMENT.** | No 0  Yes, available and observed 1  Yes, reported available but not observed 2 |  |
| **1710** | Have any provider(s) of blood transfusion services received any training in the appropriate use of blood and safe transfusion practices in the last two years? | No 0  Yes 1 |  |
| **END OF INTERVIEW. THANK THE RESPONDENT FOR THEIR TIME.** | | | |

1. Non-relevant questions and sections eliminated [↑](#footnote-ref-1)
2. [↑](#footnote-ref-2)
